# A Candidate Gene Association Study Identifies *DAPL1* as a Female-Specific Susceptibility Locus for Age-Related Macular Degeneration (AMD)

**DOI:** 10.1007/s12017-015-8342-1

**Published:** 2015-02-14

**Authors:** Felix Grassmann, Ulrike Friedrich, Sascha Fauser, Tina Schick, Andrea Milenkovic, Heidi L. Schulz, Claudia N. von Strachwitz, Thomas Bettecken, Peter Lichtner, Thomas Meitinger, Nicole Arend, Armin Wolf, Christos Haritoglou, Guenther Rudolph, Usha Chakravarthy, Giuliana Silvestri, Gareth J. McKay, Sandra Freitag-Wolf, Michael Krawczak, R. Theodore Smith, John C. Merriam, Joanna E. Merriam, Rando Allikmets, Iris M. Heid, Bernhard H. F. Weber

**Affiliations:** 1 Institute of Human Genetics, University of Regensburg, Franz-Josef-Strauss-Allee 11, 93053 Regensburg, Germany; 2 Department of Ophthalmology, University Hospital of Cologne, 53127 Cologne, Germany; 3 EyeCentre Southwest, 70563 Stuttgart, Germany; 4 Max Planck Institute of Psychiatry, 80804 Munich, Germany; 5 Institute of Human Genetics, Helmholtz Zentrum Munich, 85764 Neuherberg, Germany; 6 Institute of Human Genetics, Technical University Munich, 81675 Munich, Germany; 7 University Eye Hospital, Ludwig-Maximilians-University, 80336 Munich, Germany; 8 Centre for Experimental Medicine, Queen’s University of Belfast, Belfast, BT12 6BA Northern Ireland, UK; 9 Centre for Public Health, Queen’s University of Belfast, Belfast, BT12 6BA Northern Ireland UK; 10 Institute of Medical Informatics and Statistics, Christian-Albrechts University, 24105 Kiel, Germany; 11 Department of Ophthalmology, Columbia University, New York, NY 10032 USA; 12 Department of Ophthalmology, New York University School of Medicine, New York, NY 10016 USA; 13 Department of Pathology and Cell Biology, Columbia University, New York, NY 10032 USA; 14 Department of Genetic Epidemiology, University of Regensburg, 93053 Regensburg, Germany

**Keywords:** Age-related macular degeneration, Death-associated protein-like 1, DAPL1, Canonical *DAPL1* isoforms, Genetic association study

## Abstract

Age-related macular degeneration (AMD) is the leading cause of blindness among white caucasians over the age of 50 years with a prevalence rate expected to increase markedly with an anticipated increase in the life span of the world population. To further expand our knowledge of the genetic architecture of the disease, we pursued a candidate gene approach assessing 25 genes and a total of 109 variants. Of these, synonymous single nucleotide polymorphism (SNP) rs17810398 located in death-associated protein-like 1 (*DAPL1*) was found to be associated with AMD in a joint analysis of 3,229 cases and 2,835 controls from five studies [combined *P*_ADJ_ = 1.15 × 10^−6^, OR 1.332 (1.187–1.496)]. This association was characterized by a highly significant sex difference (*P*_diff_ = 0.0032) in that it was clearly confined to females with genome-wide significance [*P*_ADJ_ = 2.62 × 10^−8^, OR 1.541 (1.324–1.796); males: *P*_ADJ_ = 0.382, OR 1.084 (0.905–1.298)]. By targeted resequencing of risk and non-risk associated haplotypes in the *DAPL1* locus, we identified additional potentially functional risk variants, namely a common 897-bp deletion and a SNP predicted to affect a putative binding site of an exonic splicing enhancer. We show that the risk haplotype correlates with a reduced retinal transcript level of two, less frequent, non-canonical *DAPL1* isoforms. *DAPL1* plays a role in epithelial differentiation and may be involved in apoptotic processes thereby suggesting a possible novel pathway in AMD pathogenesis.

**Electronic supplementary material:**

The online version of this article (doi:10.1007/s12017-015-8342-1) contains supplementary material, which is available to authorized users.

## Introduction

Age-related macular degeneration (AMD) is a common condition of complex etiology with major risk factors including age, gender, smoking, ethnicity and genetics (Zarbin et al. 2014). While AMD ultimately represents the primary cause of blindness in developed countries (Resnikoff et al. 2004), its early form is less severe and characterized by the mere presence of drusen and pigmentary abnormalities in the macular area of the retina (Sarks et al. 1999). Late stage AMD manifests as choroidal neovascularization and/or geographic atrophy and is associated with irreversible central visual loss (Ferris et al. 2005; Zarbin et al. 2014).

Genetic predisposition plays an important role in AMD and is estimated to contribute up to 70 % of the disease risk (Seddon et al. 2005). To date, two major and several minor to moderate AMD susceptibility loci have been identified with per allele odds ratios (OR) ranging from 1.3 to 3.4 (Fritsche et al. 2013). Of note, many of these loci suggest an involvement of inflammatory processes and impaired complement activation in AMD pathogenesis (Klein et al. 2005; Gold et al. 2006; Yates et al. 2007; Hughes et al. 2007; Fagerness et al. 2009), a fact that has raised major interest in novel therapeutic approaches to address progression of the disease (Troutbeck et al. 2012).

Genetic variants associated with complex diseases are usually identified by high-throughput genome-wide association studies of large numbers of cases and controls (Fu et al. 2013). However, candidate gene studies with similar sample sizes normally have greater statistical power to detect genetic disease associations (Amos et al. 2011), especially for genes not covered efficiently by commercially available genotyping platforms (Wilkening et al. 2009).

In this study, we aimed to expand our current knowledge of the genetic architecture of AMD pathogenesis, following a candidate gene approach. In a well-powered case–control study, we screened 109 haplotype tagging variants in 25 genes for an association with late stage AMD. Attempts to replicate any positive findings in over 4,000 individuals from four previous studies revealed that variation in the death-associated protein-like 1 (*DAPL1*) gene is significantly associated with AMD. Importantly, this association is restricted to females and the variants of interest correlate with altered transcription levels of specific retinal isoforms of the *DAPL1* gene.

## Results

### Association of 109 SNPs in 25 Candidate Genes with Late Stage AMD

We first selected 25 genes and 109 haplotype tagging single-nucleotide polymorphisms (SNPs) for an initial analysis of 710 late stage AMD cases and 612 controls (GER1) (Table 1; Supplementary Tables S1, S2). Criteria for candidate gene selection included one or a combination of the following: (1) causative involvement of the gene in phenotypically related retinopathies, (2) known gene function compatible with suspected AMD pathogenesis, (3) specific or predominant gene expression in cellular sites of primary AMD pathology, i.e., the photoreceptor/retinal pigment epithelium (RPE)/choroid complex. All SNPs were tested for significant deviation from Hardy–Weinberg equilibrium (*P* < 0.05) in all controls and in female and male controls separately. This identified three SNPs (*RGR:*rs2279227, rs4620343 and *TRPM3:*rs3812532) which were subsequently excluded from further analyses. Association tests adjusted for age and sex revealed a nominally significant association using logistic regression between AMD and three SNPs (*DAPL1*: rs17810398:C>T, *P* = 0.016; *RP1*: rs9643828:T>C, *P* = 0.037; *CST3*: rs2424577:C>T, *P* = 0.028) (Supplementary Table S2).

**Table 1:** Summary characteristics of participating study populations

Stage	Study	Number of individuals						Study type	Mean age (SD) [years] in		
		Cases	GA^a^	NV^b^	GA&NV^c^	Controls	Total		Cases	Controls	Fraction male (%)
1	GER1	710	161	423	126	612	1,322	Case/control	78.81 (6.64)	78.21 (5.28)	36.99
2	GER2	996	216	535	245	645	1,641	Case/control	76.15 (7.32)	73.05 (8.34)	37.72
2	US	681	165	516	0	367	1,048	Case/control	79.08 (8.48)	74.57 (7.10)	39.79
2	UK	300	38	252	10	183	483	Case/control	78.45 (9.75)	74.53 (8.91)	34.78
2	COL	542	55	459	28	1,028	1,570	Population based	75.49 (7.11)	69.51 (5.82)	42.93
1 + 2	ALL	3,229	635	2,185	409	2,835	6,064	Mixed	77.68 (7.78)	73.14 (7.49)	39.36

^a^Geographic Atrophy

^b^Neovascular AMD

^c^Individuals with both GA and NV in either the same eye or in different eyes

### Replication of Three Nominally Significant AMD-Associated Candidate Gene Variants

The three SNPs with a nominally significant AMD association were genotyped in an independent German replication sample consisting of 996 late stage AMD cases and 645 controls (GER2). The disease association could be confirmed only for rs17810398 (*P* = 0.0014), a synonymous SNP in the coding sequence of the death-associated protein-like 1 (*DAPL1*) gene (Table 2). Analysis of this SNP in three other studies (681/367 late stage cases/controls from US, 300/183 from UK and 542/1028 from Cologne, Table 1) yielded consistent results (Table 2). Combined analyses of the 3,229 cases and 2,835 controls yielded a P value of 1.15 × 10^−6^ after adjustment for age, sex and study (Table 2). Given that 106 tests were performed, this result is significant at a significance level of 1.2 × 10^−4^ after Bonferroni correction. The risk allele frequencies were similar in all four studies (13.3–14.3 % in cases; 10.3–12.4 % in controls) and the per allele OR were consistent in direction and magnitude (1.177 ≤ OR ≤ 1.530) with no indication for heterogeneity (*I* ^2^ = 0).

**Table 2:** Association between AMD and rs17810398:C>T and rs17810816:A>G in five independent studies computed by logistic regression adjusted for covariates (*N* = 3,229 cases and 2,835 controls)

Sample	Number of individuals (females/males)		All				Females				Males			
			MAF^a^		*P*_ADJ_^c^	OR (95 % CI)^b,c^	MAF^a^		*P*_ADJ_^d^	OR (95 % CI)^b,d^	MAF^a^		*P*_ADJ_^d^	OR (95 % CI)^b,d^
	Cases	Controls	Cases	Controls			Cases	Controls			Cases	Controls		
rs17810398														
GER1	710 (455/255)	612 (378/234)	0.133	0.106	0.028	1.311 (1.031–1.673)	0.134	0.098	0.026	1.415 (1.046–1.930)	0.131	0.118	0.501	1.147 (0.770–1.714)
GER2	996 (670/326)	645 (352/293)	0.143	0.109	1.39E − 03	1.448 (1.157–1.823)	0.150	0.090	5.64E − 05	1.891 (1.396–2.598)	0.127	0.132	0.957	1.010 (0.714–1.433)
US	681 (428/253)	367 (203/164)	0.141	0.109	0.037	1.366 (1.024–1.838)	0.149	0.094	3.58E − 03	1.838 (1.233–2.804)	0.127	0.128	0.869	0.964 (0.626–1.497)
UK	300 (193/107)	183 (122/61)	0.145	0.104	0.047	1.530 (1.013–2.352)	0.155	0.102	0.044	1.701(1.026–2.900)	0.126	0.107	0.560	1.238 (0.613–2.611)
C OL	542 (314/228)	1,028 (582/446)	0.137	0.124	0.199	1.177 (0.917–1.508)	0.131	0.126	0.234	1.235 (0.870–1.746)	0.145	0.122	0.472	1.139 (0.796–1.623)
ALL^e^	3,229 (2,060/1,169)	2,835 (1,637/1,198)	0.140	0.113	1.15E − 06	1.332 (1.187–1.496)	0.144	0.105	2.62E − 08	1.541 (1.324–1.796)	0.131	0.124	0.382	1.084 (0.905–1.298)
rs17810816														
GER1	710 (455/255)	612 (378/234)	0.184	0.134	8.49E − 04	1.435 (1.163–1.778)	0.183	0.124	0.0,021	1.526 (1.169–2.008)	0.185	0.152	0.147	1.292 (0.916–1.832)
GER2	996 (670/326)	645 (352/293)	0.179	0.145	4.92E − 03	1.335 (1.093–1.636)	0.184	0.122	1.47E − 04	1.704 (1.300–2.256)	0.170	0.172	0.854	0.972 (0.716–1.322)
US	681 (428/253)	367 (203/164)	0.180	0.144	0.021	1.370 (1.051–1.797)	0.187	0.132	0.0,085	1.622 (1.139–2.346)	0.167	0.159	0.683	1.089 (0.725–1.646)
UK	300 (193/107)	183 (122/61)	0.166	0.149	0.497	1.144 (0.779–1.693)	0.168	0.145	0.390	1.231 (0.770–1.998)	0.162	0.158	0.944	0.976 (0.503–1.938)
COL	542 (314/228)	1,028 (582/446)	0.188	0.159	0.072	1.223 (0.981–1.522)	0.175	0.156	0.139	1.257 (0.926–1.701)	0.205	0.162	0.242	1.209 (0.879–1.660)
ALL^e^	3,229 (2,060/1,169)	2,835 (1,637/1,198)	0.180	0.148	1.76E − 07	1.318 (1.188–1.462)	0.181	0.137	2.68E − 08	1.471 (1.285–1.687)	0.179	0.162	0.141	1.129 (0.961–1.326)

^a^Minor allele frequency

^b^Odds ratio (OR) and 95 % confidence intervals (95 % CI)

^c^Adjusted for age and sex

^d^Adjusted for age

^e^Analyses were additionally adjusted for study

### Imputation and Replication of Genetic Variants at the DAPL1 Locus

**Fig. 1:**
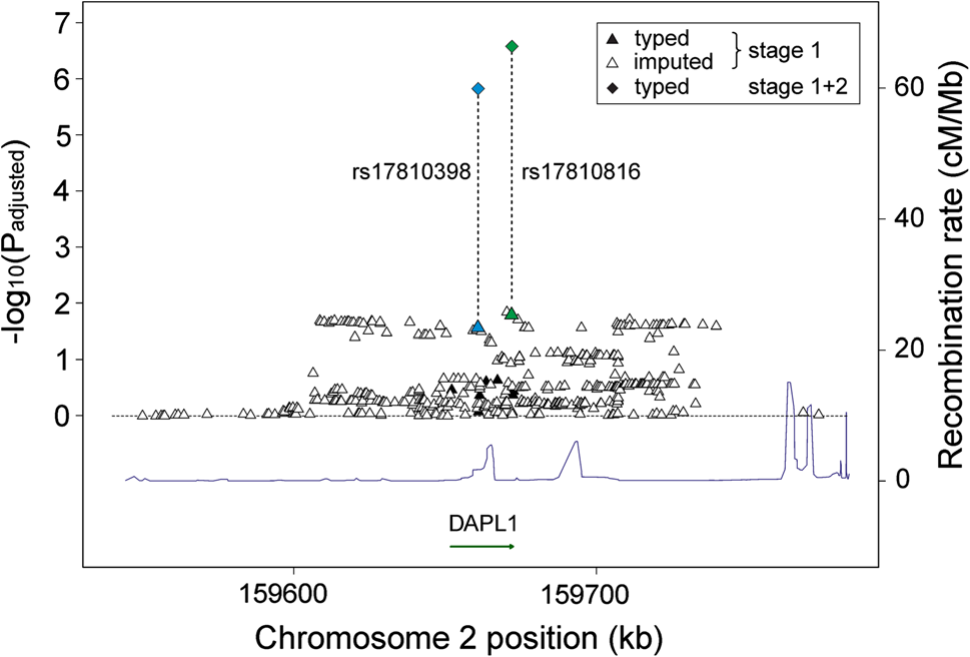
Association with AMD of imputed and typed variants at the *DAPL1* locus. Association signals of markers are shown by the log-*P* value from a logistic regression model (additive model adjusted for age and sex; *y*-axis) and are plotted against their physical position (*x*-axis). Stage 1 results (GER1 sample) are marked by filled (genotyped) and open triangles (imputed). Association signals of rs17810398 and rs17810816 in the pooled samples (3,229 cases and 2,835 controls) are indicated by *blue* and *green diamonds*, respectively

We next imputed the genotypes of 20,422 additional SNPs around rs17810398 in the GER1 study based on 8 tagging SNPs at the DAPL1 locus. After quality control, 517 SNPs were included in the analysis and association signals (*P*_ADJ_ < 0.05) were obtained that were confined to a 154-kb region devoid of any gene other than *DAPL1* (Fig. 1; Supplementary Table S3). Forty-eight imputed SNPs revealed an association more significant than rs17810398 (*P*_ADJ_ = 0.027), with a minimum *P*_ADJ_ of 0.014 at rs74923781 and its perfect proxy rs17810816:A>G (*r* ^2^ = 1 based on 1,000 Genomes CEU samples) in *DAPL1* intron 4. De novo genotyping of rs17810816 gave consistent association in terms of its direction in all four replication studies with a nominal significance (*P*_ADJ_ ≤ 0.05) attained in GER2 and US. There was no indication of heterogeneity (*I* ^2^ = 0). The combined analysis yielded a *P*_ADJ_ of 1.76 × 10^−7^ after adjustment for age, sex and study (Table 2; Fig. 1). Linkage disequilibrium (LD) and haplotype analysis in the GER1 study revealed rs17810398 and rs17810816 to be in moderate LD in controls (*r* ^2^ = 0.55, Supplementary Figure S1).

### Variants rs1710398 and rs17810816 Show a Female-Specific Association

**Fig. 2:**
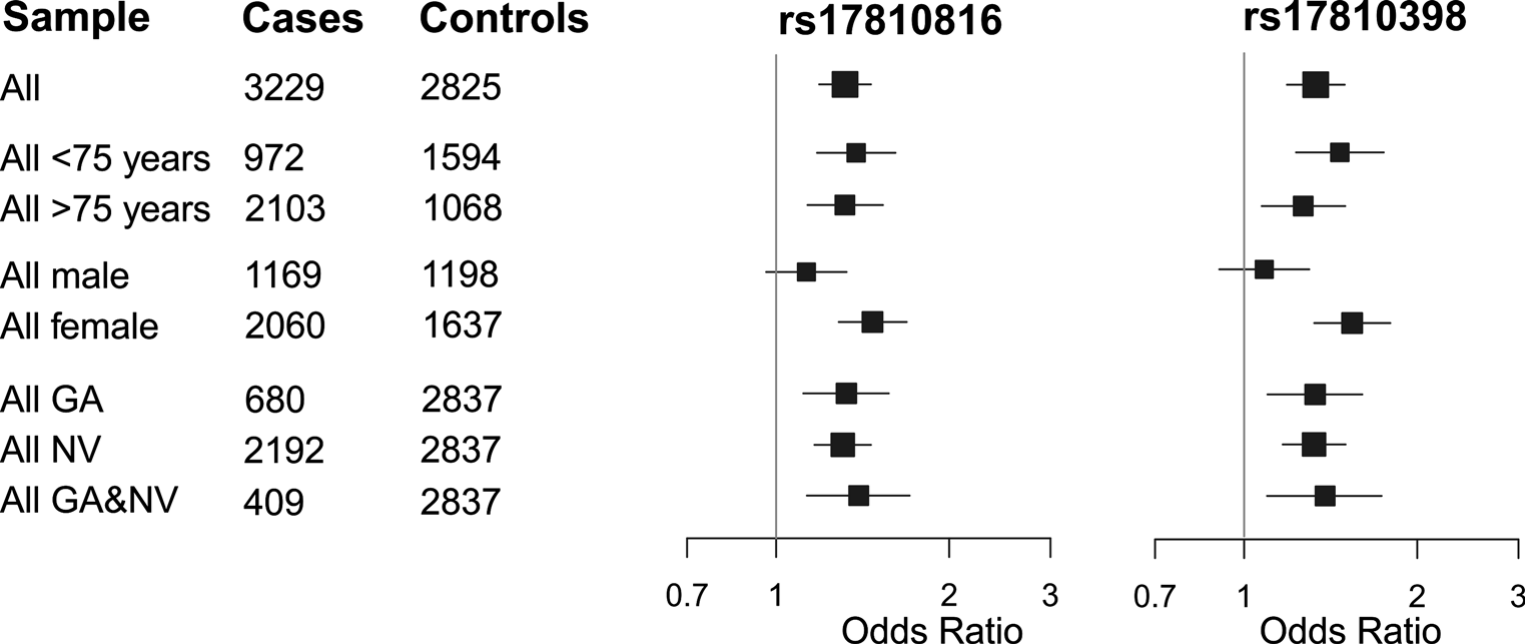
Subgroup analysis in the combined study of candidate SNPs rs17810398 and rs17810816 in the DAPL1 gene. OR and corresponding 95 % confidence intervals are given with the size of each rectangles representing the respective number of cases. AMD phenotypic subgroups comprise patients with geographic atrophy (GA), and neovascular AMD (NV) and both late stage forms (GA&NV)

Stratifying the combined analysis by phenotype, including AMD subtype and age-group, revealed no subgroup-specific association for rs17810398 or rs17810816 (Fig. 2). However, stratification by sex revealed that the association signals of both SNPs were confined to females with genome-wide significance (rs1710398: *P*_*ADJ*_ = 2.62 × 10^−8^, rs17810816: *P*_*ADJ*_ = 2.68 × 10^−8^). No AMD association was evident in males (rs1710398: *P*_*ADJ*_ = 0.382, rs17810816: *P*_*ADJ*_ = 0.141; Fig. 2, Supplementary Figure S2; Table 2). The difference between sex-specific ORs was statistically significant (rs17810398: *P*_diff_ = 0.0034, rs17810816: *P*_diff_ = 0.014) at the 5 % level and was observed in all studies analyzed (Supplementary Figure S2; Table 2). In the combined study, the minor allele frequency (MAF) of rs17810398 was lower in female controls than in male controls and higher in female cases than in male cases. A similar, albeit less pronounced effect was seen for variant rs17810816.

### DAPL1 Encodes Four Isoforms in Retina/RPE

**Fig. 3:**
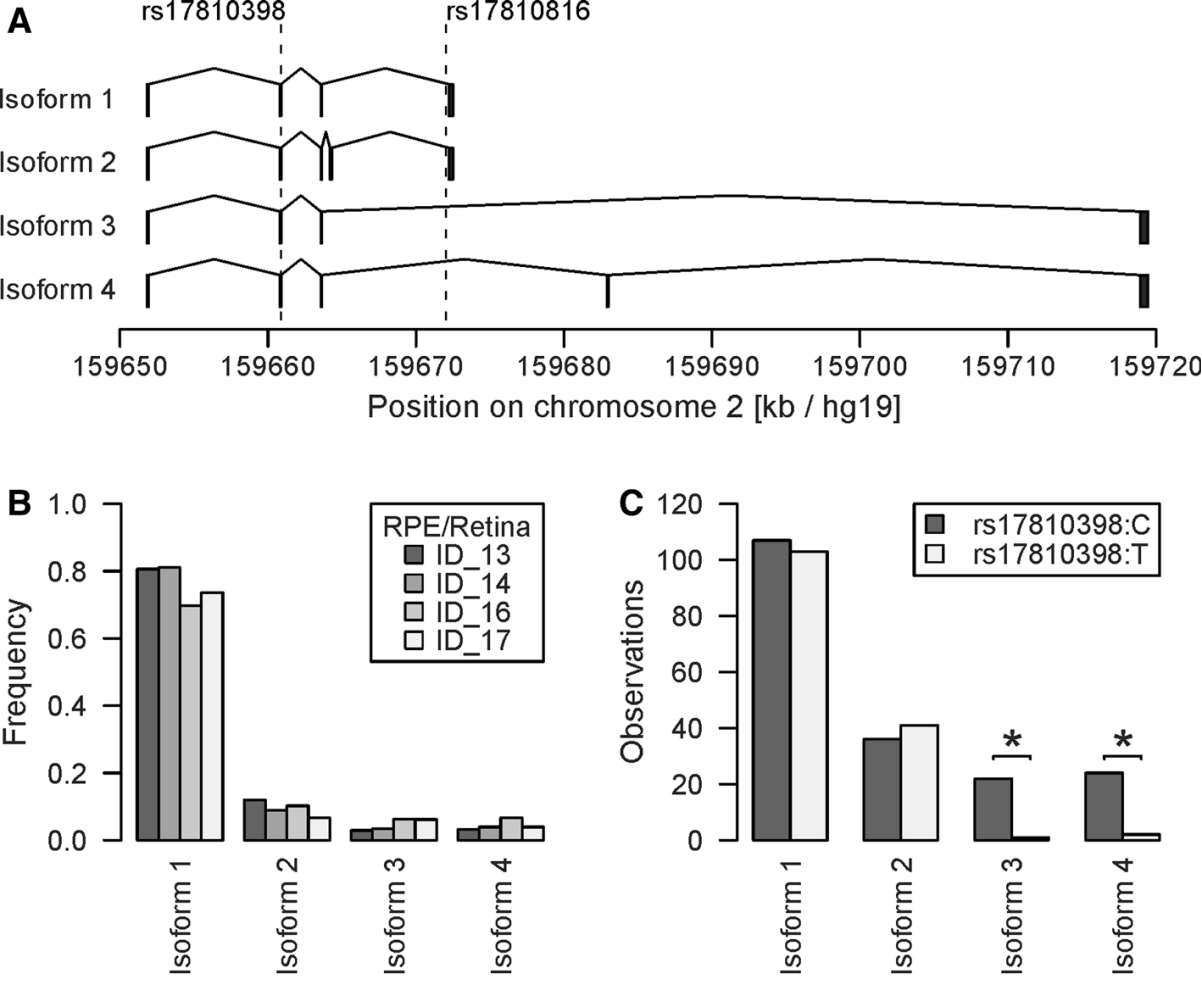
Functional consequences for isoform expression of DAPL1 variants. **a** Exon/intron structure of four frequent DAPL1 isoforms (gene orientation is from left to right). SNP positions are marked by vertical dotted lines. **b** Frequency of the four isoforms as determined from sequencing 1,200 cDNA clones that were obtained after 3′-RACE of four unrelated RPE/retina tissue samples either homozygous (ID_16, ID_17) or heterozygous (ID_13, ID_14) for the non-risk alleles of rs17810398 and rs17810816. **c** Distribution of rs17810398 alleles in heterozygous RPE/retina tissue samples ID_13 and ID_14. Statistically significant deviations from the reference transcript (i.e., isoform 1) are indicated by asterisks (*P* < 0.0001)

For expression analysis of the *DAPL1* locus, we scrutinized expressed sequence tags (EST) and identified three entries [GenBank accession numbers: DA417123 (thalamus), BG818506 (oligodendroglioma), BI016096 (lung tumor)] that suggested alternative splicing of *DAPl1* gene products. A potential correlation between rs1710398 and rs17810816 genotype and the occurrence of *DAPL1* isoforms was investigated by 3′-RACE experiments on four unrelated RPE/retina tissue samples, two of which were homozygous (ID_16 and ID_17) for the non-risk alleles and two of which were heterozygous (ID_13 and ID_14) for the risk alleles. After plasmid cloning of PCR products, we sequenced 1,200 cDNA clones and identified a total of 24 specific DAPL1 isoforms four of which (referred to as isoforms 1–4, Supplementary Figure S3) were consistently found in all samples. The most abundant isoform 1 (65–77 % over all samples) corresponded to the *DAPL1* reference sequence (NM_001017920). Isoform 2 (6–12 %) and 3 (3–6 %) had not been reported before, whereas isoform 4 (3–6 %) matched EST BG818506 (Fig. 3a, b). Sequences corresponding to DA417123 and BI016096 were not detected in the RPE/retina RNA samples. RT-PCR analysis confirmed the expression of isoforms 1–4 in human tissues with isoform 4 likely being specific for RPE/retina (Supplementary Figure S4).

### Resequencing of Candidate Regions at the DAPL1 Locus

In a search for additional risk variants at the extended *DAPL1* locus, we resequenced over 10 kb of intronic/exonic sequences in each of 12 probands homozygous for AMD risk alleles rs17810398:T and rs17810816:G and eight probands homozygous for AMD non-risk alleles rs17810398:C and rs17810816:A (Supplementary Figure S3; Supplementary Table S4). Due to its extensive saturation with repeat structures, resequencing of the genomic region around *DAPL1* exon 4 of HQ179937 (isoform 4) was carried out for three individuals following sub-cloning of PCR fragments. In total, we detected 33 sequence variants (Supplementary Table S5), three of which (rs75277023:G>A, rs6146986, and rs144087548:A>T) were in strong LD with rs17810398 and rs17810816 (*r* ^2^ in controls > 0.9, Supplementary Figure S1). Variants rs6146986 and rs144087548 were of particular interest as the minor allele of the former represents a common 878-bp deletion in *DAPL1* intron 2, and the latter was predicted to affect a putative binding site of an exonic splicing enhancer (serine/arginine-rich splicing factor 1, SRSF1), 24-bp upstream of the most 3′ exon shared by isoforms 3 and 4 (Supplementary Figure S3). Genotyping of rs6146986 and rs144087548 in the GER1 study confirmed their strong LD with rs17810398 (rs6146986: *r* ^2^ = 0.93) and rs17810816 (rs144087548: *r* ^2^ = 0.77) (Supplementary Figure S1; Table 3). Additional cDNA resequencing of eight RPE/retina tissues heterozygous for rs17810398 did not reveal additional coding variants (Supplementary Table S6).

**Table 3:** Association results in the GER1 study for four functional candidate SNPs in *DAPL1*

SNP	Position on chr 2 (bp/hg19)	Major allele	Minor allele	MAF^b^		Odds ratio (95 % CI)	*P* ^a^	R^2c^
				Cases (%)	Controls (%)			
rs17810398	159,660,870	C	T	13.3	10.5	1.314 (1.033–1.680)	0.027	Ref.
rs6146986	159,661,997–159,662,874	–	878-bp deletion	13.5	10.7	1.310 (1.033–1.667)	0.027	NA
rs17810816	159,671,992	A	G	18.4	13.4	1.435 (1.163–1.778)	8.49 × 10^−4^	0.866
rs144087548	159,718,894	A	T	17.4	13.8	1.296 (1.047–1.609)	0.018	0.735

^a^ *P* from logistic regression adjusted for age and sex

^b^MAF: minor allele frequency calculated in 710 cases or 612 controls

^c^R2 to top variant based on CEU samples from the 1,000 genomes project

### AMD-Associated Variants are Correlated with Differential Expression of DAPL1 Isoforms

**Fig. 4:**
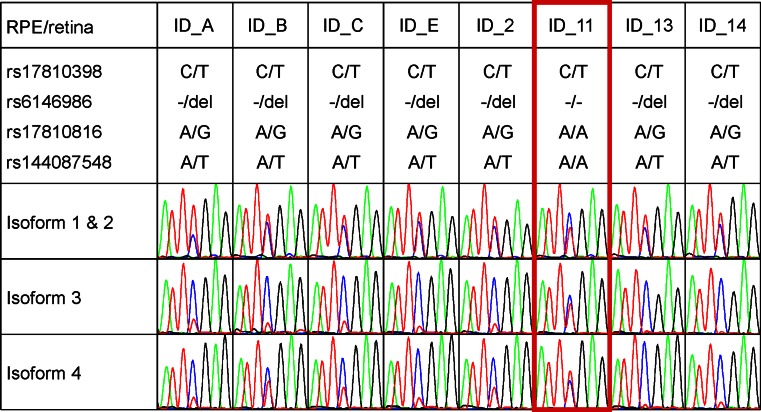
Semi-quantitative cDNA sequencing of eight RPE/retina tissue samples heterozygous for synonymous coding SNP rs17810398:C>T. The chromatograms of the variant nucleotide at rs17810398 flanked by ±3 bp are shown for isoforms 1 & 2, 3, and 4. Isoforms were specifically amplified by three different exon-spanning primer combinations. Genotypes of the four candidate variants are given above the chromatograms. The sample heterozygous for rs17810398 but homozygous for the non-risk alleles of rs6416986, rs17810816, and rs144087548 is highlighted in *red*

Samples heterozygous for rs17810398 (ID_13 and ID_14) were characterized by a significantly different abundance of non-risk and risk isoforms 3 and 4 (*P* < 10^−4^). This was not the case for isoforms 1 and 2 (Fig. 3c; Supplementary Table S7). *DAPL1* isoform expression in RPE/retina was further evaluated in vivo by semi-quantitative cDNA sequencing (Fig. 4). Of 39 unrelated RPE/retina tissues available, seven were heterozygous for AMD-associated variants rs17810398, rs6146986, rs17810816, and rs144087548. In agreement with our 3′-RACE data, these samples revealed differential expression of isoforms 3 and 4 but not isoforms 1 and 2 (Fig. 4). Interestingly, sample ID_11 was heterozygous for rs17810398, but homozygous for the non-risk alleles of rs6146986, rs17810816, and rs144087548. In this sample, expression intensities of isoform 3 and 4 alleles were equal excluding rs17810398 as a functional variant involved in the differential expression of isoforms 3 and 4. This leaves rs6146986, rs17810816, and rs144087548 or an as yet unknown but correlated variant as the truly functional risk variant at the *DAPL1* locus.

## Discussion

Here, we provide evidence that *DAPL1* is an AMD-associated gene and that its disease association is female-specific. To our knowledge, this is a first study reporting a sex-specific genetic association with AMD at a genome-wide significance level. Although lead SNPs rs17810398 and rs17810816 have been imputed into large GWAS data sets, neither variant has been identified before as AMD-associated (Fritsche et al. 2013). This is likely due to the female specificity of the association as male/female ratios in multi-center GWAS tend to differ greatly between cohorts thereby potentially leading to reduced power. Behrens et al. (2011) have methodologically shown that gender-stratified analyses greatly increase power to detect gender-specific effects. Additionally, we have also observed the female-specific association in our population-based sample (COL study) after adjusting the analysis for age. This further indicates that different study types increase the heterogeneity and therefore may lead to decreased power to detect this association.

We also considered the possibility that age is a confounding factor in our study since (1) females are slightly older than males and (2) cases are older than controls. If any of the variants would be correlated with longevity, this could potentially confound our analysis. However, we found no evidence for a correlation between either SNP or age, neither in cases, controls, females nor males separately or analyzed jointly (*P* > 0.05). Additionally, we note that logistic regression analyses were adjusted for age in our analyses. Furthermore, two SNPs at the *DAPL1* locus (rs9869 and rs10497199) were investigated in a recent study by Flachsbart et al. 2010. For these two variants, the authors found no association with longevity (*P* > 0.5). Variant rs9869 is weakly linked to markers rs17810398 (*r* ^2^ = 0.13) and rs17810816 (*r* ^2^ = 0.1), while rs10497199 is independent of either variant (*r* ^2^ < 0.1). Taken together, these findings have led us to exclude a confounding effect of age in our analysis.

The observed association in the present study could eventually be explained by a population substructure in our cases or controls from the UK or US. Although we cannot definitely exclude such a possibility, it is of note that frequency and effect sizes of the two DAPL1 risk variants rs17810398 and rs17810816 (in males, females and jointly) observed in the UK and US study are similar to the frequencies observed in the combined German cohort (Table 2). The German samples derive from a genetically homogenous population from a small area in southern Germany. Homogeneity was estimated previously from genome-wide data available for a subset of cases (Fritsche et al. 2013). From these data, we conclude that the US and UK study primarily consists of Caucasians which are genetically similar to the German cohort and, if at all, population substructure may only exert a minor effect on study outcome.

Although *DAPL1* is evolutionarily conserved, only little is known about its function. It has been shown to be abundantly expressed in the retina/RPE transcriptome (Schulz et al. 2004) as well as in epidermis, esophageal epithelium, and tongue epithelium where it appears to be involved in the early stages of stratified epithelial differentiation (Sun et al. 2006). Based upon strong amino acid sequence similarities, *DAPL1* has also been connected to the death-associated protein (DAP), a basic, proline-rich protein of 15-kD molecular weight that acts as a positive mediator of programmed cell death upon induction by interferon-gamma (Deiss et al. 1995). Clarification of the cellular function of DAPL1 in the RPE/retina is required to allow more detailed insight into this novel pathway of AMD pathogenesis.

We have shown that *DAPL1* is present in a multitude of correctly spliced isoforms, two of which, isoforms 3 and 4, were specifically down-regulated in the presence of AMD-associated alleles. Although we could not identify the causative variant at the *DAPL1* locus, we excluded lead SNP rs17810398 as the presence of the T-risk allele in one patient (ID_11) had no influence on the transcript levels of isoform 3 or 4. Notably, the unique C-terminus of isoform 4 encodes two potential transmembrane domains with significant homology to the rhodopsin-like G protein-coupled receptor (GPCR) family. Another member of the GPCR family, the G protein-coupled estrogen receptor 1 (GPER) plays a role in intracellular signaling following estrogen binding and could provide a useful lead when searching for factors involved in sex-dependent AMD risk. While at present we cannot explain the gender-specificity of the association with *DAPL1*, our results provide a starting point at a molecular level to investigate why AMD is more frequent in women than in men (Owen et al. 2012).

Taken together, we investigated 25 gene loci of interest to AMD pathology and excluded all but one from being disease-associated. Our data implicate *DAPL1* as a novel gene involved in AMD pathology although the cellular functions of this gene and of its various differentially spliced transcripts remain elusive. Our study revealed a correlation between risk variants at rs17810398 and rs17810816 on the one hand and expression levels of *DAPL1* isoforms 3 and 4 on the other, the latter being specifically expressed in RPE/retina tissue. We also reported a significant sex difference of the effect of *DAPL1* where only females showed an association signal at this locus. Although speculative at present, this sex difference may be explained by a role of *DAPL1* variants in sex-specific signaling processes. Our findings add another piece to the puzzle of the genetic architecture of AMD, which, once completed, should allow refined identification of individuals at risk for this disease.

## Methods

### Subjects

Five independent studies were included in our study comprising a total of 3,053 unrelated Caucasian patients with clinically documented late stage AMD (cases) and 2,738 unrelated age and individuals with comparable age range and ethnicity without signs of macular disease (controls) (Table 1). All data were available for analysis at the analysis center in Regensburg. Discovery study GER1 (stage 1) included 710 AMD patients and 612 controls from the University Eye Clinic of Würzburg (Germany). The four replication studies (Stage 2) comprised (1) 996 AMD patients and 645 controls from the University Eye Clinics in München, Tübingen and Würzburg (Germany) (GER2); (2) 681 AMD patients and 367 controls from Columbia University (New York, USA) (US); (3) 300 AMD patients and 183 controls from the Royal Victoria Hospital (Belfast, UK) (UK); and (4) 542 AMD patients and 1,028 controls from the Department of Ophthalmology at the University Hospital Cologne, Germany (COL). Cases and controls were examined by trained ophthalmologists. Stereo fundus photographs were graded according to standardized classification systems as described previously (Grassmann et al. 2012). The study was conducted at all sites in strict adherence to the tenets of the Declaration of Helsinki and was approved by the respective Ethics Committees at the University Eye Clinics of Würzburg, München and Tübingen, by the Institutional Review Board at Columbia University, by the Research Ethics Committee of Queen’s University Belfast and by the local Ethics Committee in Cologne.

### Genotyping

Genomic DNA was extracted from peripheral blood leukocytes according to established protocols. Genotyping of SNPs was carried out by direct sequencing, TaqMan SNP genotyping (Applied Biosystems, Foster City, USA) or by primer extension of multiplex PCR products and subsequent allele detection by matrix-assisted laser desorption/ionization time of flight (MALDI-TOF; Sequenom, San Diego, USA). Direct sequencing was performed with the Big Dye Terminator Cycle sequencing kit version 1.1 (Applied Biosystems, Foster City, U.S.A.) according to the manufacturer’s instructions. Reactions were analyzed with an ABI Prism 3130xl sequencer (Applied Biosystems). TaqMan pre-designed SNP genotyping assays (Applied Biosystems) were used according to the manufacturer’s instructions. The rs144087548 variant was genotyped by polymerase chain reaction (forward primer: 5′-CGC AGA CAT GAT GCT GGG GGT-3′; reverse primer: 5′-ACA TGC AAG ACG GGG AAT TGA-3′) followed by *HpyCH4III* digestion (New England Biolabs, Ipswich, USA) and restriction fragment length analysis. All SNPs showed high genotyping quality with an average call rate >98 % in each of the five case–control samples.

### Statistical Methods

#### Discovery Study

We excluded three SNPs [rs2279227 (*RGR*), rs4620343 and rs3812532 (*TRPM3*)], each with significant deviation from Hardy–Weinberg equilibrium (HWE, *P* ≤ 0.05) in the control group of the discovery sample. SNP association analysis was carried out by logistic regression adjusted for age and sex. All analyses modeled an additive genetic effect and the genotype was coded as the number of alleles present at a given variant (i.e., 0, 1 or 2).

#### Replication Studies and Combined Analysis

All SNPs were in HWE (*P* > 0.05). We used the same tests for SNP association analysis as in the discovery study. We also combined the individual data from all five studies and also adjusted the respective analyses by study center (coded as factors). The *I* ^2^ measure was computed to measure between-study heterogeneity. We also conducted sex-stratified analyses for each study separately and for all study samples combined. Sex differences were asses for statistical significance using a *t* test derived from sex-specific beta estimates and corresponding standard errors.

All reported *P* values were two-sided except where noted otherwise. All SNP association analyses were carried out with R (v3.0.1, http://R-Forge.R-project.org/). To allow a more detailed inspection of the genomic region of interest, measures of LD were calculated using R package snp.plotter (Luna and Nicodemus 2007).

### Imputation of SNPs

Prior to imputation, 8 tag SNPs in *DAPL1* were phased in the GER1 study individuals using SHAPEIT2 (Delaneau et al. 2013). Then, untyped SNPs were imputed with IMPUTE2 (Howie et al. 2009) using the 1,000 Genomes Phase I integrated haplotypes (release 20110521) as reference panel. After the exclusion of SNPs with imputation quality (“info”) <0.5, the genotype probabilities (dosages) of the remaining SNPs were also analyzed by logistic regression in R, using an additive model adjusted for age and sex.

### Genomic Resequencing

Genomic resequencing was done for regions of interest defined by the presence of certain gene elements (putative promoter, coding exons of transcripts NM_001017920.2, HQ179935, HQ179936, and HQ179937) or conserved elements based upon the “46-Way Most Cons” track of the UCSC genome browser, NCBI Build 37/hg19. Regions within extensive repeat structures were excluded (Supplementary Figure S3). Resequencing primers are listed in Supplementary Table S4.

### Prediction of Functional Impact of Risk Variants

The functional impact AMD-associated SNPs (with known dbSNP ID) on RNA processing as well as protein sequence, structure and function was predicted using the web-based “SNP Function Prediction” tool implemented in the “SNPinfo Web Server” (http://snpinfo.niehs.nih.gov/index.html) (Xu and Taylor 2009). For newly identified SNPs, we used ESEfinder 3.0 to predict the effect of a given SNP allele on putative exonic splicing enhancers **(** http://rulai.cshl.edu/cgi-bin/tools/ESE3/esefinder.cgi) (Cartegni et al. 2003).

### Characterization of Major Splice Variants of DAPL1 in Human Retina/RPE

To determine major splice variants and functional polyadenylation sites, 3′ rapid amplification of cDNA ends (3′-RACE) experiments were conducted. RNAs from RPE/retina tissues that were either heterozygous (ID_13 and ID_14) or homozygous (ID_16 and ID_17) for the non-risk rs17810398:C allele were isolated by RNeasy Mini Kit followed by DNAse I treatment (QIAGEN, Hilden, Germany). 3′-RACE was conducted with the FirstChoice RLM-RACE Kit (Applied Biosystems/Ambion, Austin, USA) according to the manufacturer’s instructions. Forward primers for first and second (nested) PCR were 5′-GCA CTG GCA CACG CTA TG-3′ and 5′-TTG GCA CCT TGG AAA GAC ATA CC-3′, respectively. Amplified RACE products were ligated into the pGEM-T vector (Promega, Madison, USA). PCR products were obtained with M13 forward and M13 reverse primers from a total of 1,200 clones. Of these, 597 clones were sequenced; the remaining 603 could unequivocally be assigned to *DAPL1* isoform 1 (NM_001017920.2, HQ179934) by visual gel inspection. The sequences of isoforms 2–6 were submitted to GenBank (HQ179935, HQ179936, HQ179937, HQ179938, HQ179939).

### Expression Analysis and Semi-quantitative Resequencing

Eight RPE/retina tissues with risk variant genotypes as given in Fig. 3 and Supplementary Table S6 were used as templates to amplify isoform-specific PCR products with forward primer 5′-GCA CTG GCA CAC GCT ATG-3′ and the isoform-specific reverse primers 5′-CGA GGC TGC TGA ATA ATG TAG-3′ (isoform 1 & 2), 5′-TCT GGA TCC TCT GAG CTT CTT CTC-3′ (isoform 3) or 5′-CTG GAT CCT CTG AGC TTC TTG TGT-3′ (isoform 4), followed by sequencing with the forward primer. Primers for the *GUSB* gene were 5′-ACT ATC GCC ATC AAC AAC ACA CTC ACC-3′ and 5′-GTG ACG GTG ATG TCA TCG AT-3′. For tissue samples, sex was determined with fluorescence-based PCR analysis of the homologous, X- and Y-linked genes *AMELX* and *AMELY* as described in Sullivan et al. (1993).

## Electronic supplementary material

Below is the link to the electronic supplementary material.

Supplementary material 1 (PDF 1193 kb)

Supplementary Figure S1. Linkage disequilibrium (LD) map of the *DAPL1* gene locus. SNP positions are indicated by vertical/diagonal lines. **A.** Values of r² are indicated by coloring (white, low r²; black, high r²). **B.** Values of D’ are indicated by coloring (white, low D’; black, high D’). (TIFF 537 kb)

Supplementary Figure S2. Sex-specific analysis in the combined study of candidate SNPs rs17810398 and rs17810816 in the *DAPL1* gene. Odds ratios and corresponding 95% confidence intervals are given with the size of each rectangle representing the respective number of cases. Late stage sub-phenotypes were combined. (TIFF 818 kb)

Supplementary Figure S3. Resequencing strategy of the *DAPL1* locus. A screenshot of the UCSC genome browser with default and custom tracks at position hg19:chr2: 159,650,435-159,721,003 is shown (http://genome.ucsc.edu). Track names are given above each track. From top to bottom, tracks are as follows: (1) Identified risk variants based upon discovery study (green), imputation analysis (blue; Supplementary Table S3) or resequencing (purple; Supplementary Table S5); (2) RefSeq sequence of *DAPL1* (NM_001017920.2); (3) Common *DAPL1* transcripts as identified in four RPE/retina tissue samples (isoform 1: HQ179934/NM001017920.2, isoform 2: HQ179935, isoform 3: HQ179936, isoform 4: HQ179937); (4) Exons identified in common and rare isoforms of *DAPL1*; (5) Resequenced PCR fragments (Supplementary Table S4); (6) Regions of interest based upon exon structure and conservation; (7) “46-Way Most Cons” track of the UCSC genome browser(14.03.2014); (8) UCSC RepeatMasker track (14.03.2014). (TIFF 1273 kb)

Supplementary Figure S4. RT-PCR expression analysis of *DAPL1* isoforms. All forward and reverse primers used were intron-spanning to avoid amplification of traces of genomic contamination in the mRNA preparations. Expected and observed fragment sizes were as follows: 329 bp (isoform 1), 448 bp (isoform2), 340 bp (isoform 3) and 331 bp (isoform 4). Expression analysis of housekeeping gene β-glucuronidase (GUSB; 197 bp) served as a control for first-strand cDNA integrity. (TIFF 1627 kb)
