# Supplementary material for: A Candidate Gene Association Study Identifies DAPL1 as a Female-Specific Susceptibility Locus for Age-Related Macular Degeneration (AMD)
Source: Neuromolecular Med. 2015 Feb 14;17(2):111–20. doi: 10.1007/s12017-015-8342-1 (PMC4419162; doi:10.1007/s12017-015-8342-1)
Supplement: Supplementary file 1 — Supplementary material 1 (PDF 1193 kb) [file 12017_2015_8342_MOESM1_ESM.pdf]

Supplementary Table S1. Candidate gene based analyses of 106 SNPs in the discovery sample GER1 (N_{Cases} = 710; $N_{Controls}$ = 612) and replication sample GER2(N_{Cases} = 820; $N_{Controls}$ = 547).

**Gene name**	**Symbol**	**Selection criteria***		**Chromosome band**	**# SNPs analyzed**	**Discovery Sample GER1**	**Replication Sample GER2**
		**Protein Function**	**Retinal / RPE expression**			***Pmin*********	***P**********
SH3-domain GRB2-like (endophilin) interacting protein 1	*SGIP1*	-	X	1p31.3	3	0.696	…
Retinal pigment epithelium-specific protein 65kDa	*RPE65*	X	X	1p31	4	0.059	…
ATP-binding cassette, sub-family A (ABC1), member 4	*ABCA4*	X	X	1p22	17	0.183	…
Cathepsin S	*CTSS*	X	-	1q21	2	0.742	…
G protein-coupled receptor 75	*GPR75*	-	X	2p16	2	0.780	…
c-mer proto-oncogene tyrosine kinase	*MERTK*	X	X	2q14.1	3	0.342	…
Death associated protein-like 1	*DAPL1*	-	X	2q24	8	**0.016**	**0.002**
Membrane protein, palmitoylated 4	*MPP4*	-	X	2q33.2	5	0.430	…
Retinol binding protein 1, cellular	*RBP1*	-	X	3q21-q23	4	0.444	…
Succinate receptor 1	*SUCNR1*	X	-	3q25.1	6	0.067	…
WD repeat domain 17	*WDR17*	-	X	4q34	3	0.356	…
Neuropeptide VF precursor	*NPVF*	-	X	7p21-p15	2	0.164	…
Retinitis pigmentosa 1 (autosomal dominant)	*RP1*	X	X	8q12.1	3	**0.037**	0.44
Transient receptor potential cation channel, subfamily M, member 3	*TRPM3*	-	X	9q21.11	10	0.141	…
Cadherin-related family member 1	*CDHR1*	X	X	10q23.1	6	0.197	…
Retinal G protein coupled receptor	*RGR*	X	X	10q23	2	0.096	…
Cathepsin D	*CTSD*	-	X	11p15.5	5	0.355	…
Fatty acid desaturase 3	*FADS3*	X	-	11q12-q13.1	3	0.496	…
Bestrophin 1	*BEST1*	X	X	11q12	3	0.070	…
Chromosome 11 open reading frame 48	*C11orf48*	-	X	11q12.3	2	0.149	…
Retinol dehydrogenase 12 (all-trans/9-cis/11-cis)	*RDH12*	X	X	14q24.1	1	0.247	…
Retinaldehyde binding protein 1	*RLBP1*	X	X	15q26.1	2	0.488	…
ATPase, Na+/K+ transporting, beta 2 polypeptide	*ATP1B2*	-	X	17p13.1	2	0.212	…
Neuropilin (NRP) and tolloid (TLL)-like 1	*NETO1*	-	X	18q22.2	6	0.256	…
Cystatin C	*CST3*	X	X	20p11.2	2	**0.028**	1.00

* X: present; -: not present
** Pmin: minimum *P* value from logistic regression model adjusted for age and gender for all SNPs analyzed in this region
*** *P* value from logistic regression model adjusted for age and gender

Supplementary Table S2. Association results for 106 SNPs analyzed in discovery (794 late stage cases, 612 controls)

								Minor Allele Frequency in		No. non-missing genotypes in		
Gene	SNP (dbSNP-ID)	Chr.	Position [hg19]	Major allele	Minor allele	OR	95% CI	Contr	Cases	Contr	Cases	P-Value*
SGIP1	rs1373909	1	67040875	A	G	1,018	0.872-1.188	0,420	0,424	611	710	0,821
SGIP1	rs1536112	1	67107225	A	G	1,019	0.869-1.194	0,435	0,438	608	707	0,817
SGIP1	rs6588216	1	67145847	A	G	1,010	0.850-1.200	0,277	0,280	610	708	0,910
RPE65	rs3118415	1	68894296	A	G	1,041	0.850-1.276	0,171	0,176	588	696	0,696
RPE65	rs3125895	1	68901735	G	T	1,002	0.860-1.167	0,410	0,410	608	702	0,980
RPE65	rs3790472	1	68910999	G	T	1,160	0.995-1.354	0,441	0,478	608	703	0,059
RPE65	rs436070	1	68918895	G	A	0,866	0.716-1.048	0,209	0,186	608	706	0,139
ABCA4	rs1800555	1	94463617	G	A	0,872	0.397-1.915	0,011	0,009	612	710	0,729
ABCA4	rs7537325	1	94469631	A	G	1,007	0.840-1.207	0,225	0,227	604	704	0,939
ABCA4	rs1800553	1	94473807	G	A	1,559	0.534-5.107	0,004	0,006	612	710	0,429
ABCA4	rs2275033	1	94480037	G	A	0,937	0.804-1.092	0,434	0,418	604	687	0,404
ABCA4	rs1932014	1	94488497	T	C	1,014	0.872-1.180	0,453	0,457	606	708	0,853
ABCA4	rs3789395	1	94501594	G	T	0,990	0.849-1.154	0,461	0,459	609	704	0,894
ABCA4	rs11165069	1	94504545	G	A	0,972	0.807-1.172	0,212	0,206	604	703	0,768
ABCA4	rs497511	1	94523113	T	C	1,063	0.910-1.242	0,471	0,485	611	705	0,438
ABCA4	rs549114	1	94534354	C	T	1,055	0.893-1.248	0,321	0,332	605	698	0,529
ABCA4	rs4147827	1	94548080	G	C	0,920	0.762-1.112	0,209	0,195	609	704	0,390
ABCA4	rs952499	1	94558425	A	G	0,964	0.825-1.127	0,498	0,488	611	702	0,647
ABCA4	rs950283	1	94567223	A	G	1,049	0.896-1.227	0,360	0,373	612	710	0,555
ABCA4	rs1209515	1	94571335	G	A	1,091	0.920-1.295	0,268	0,284	607	704	0,318
ABCA4	rs4147815	1	94574808	C	T	0,944	0.780-1.141	0,223	0,213	611	692	0,550
ABCA4	rs2297634	1	94576968	A	G	1,103	0.944-1.290	0,469	0,491	611	706	0,219
ABCA4	rs2184339	1	94585331	A	G	0,891	0.752-1.056	0,307	0,286	608	704	0,183
ABCA4	rs3761911	1	94588754	A	T	1,044	0.890-1.226	0,376	0,385	605	703	0,597
CTSS	rs1136774	1	150738197	A	G	1,001	0.858-1.167	0,471	0,472	611	708	0,991
CTSS	rs3754212	1	150738200	T	C	1,028	0.874-1.208	0,360	0,366	607	707	0,742
GPR75	rs805368	2	54083420	C	T	1,025	0.861-1.221	0,272	0,276	612	708	0,780
GPR75	rs805373	2	54086595	T	C	0,983	0.840-1.150	0,452	0,446	612	708	0,829
MERTK	rs7604639	2	112751928	A	G	0,930	0.792-1.092	0,395	0,378	611	709	0,373
MERTK	rs3811634	2	112754943	C	T	1,036	0.877-1.224	0,300	0,308	612	710	0,677
MERTK	rs55812028	2	112780969	A	G	0,915	0.760-1.100	0,234	0,219	612	708	0,342
DAPL1	rs2280184	2	159652231	T	G	0,924	0.790-1.081	0,416	0,399	607	706	0,322
**DAPL1**	**rs17810398**	**2**	**159660870**	**C**	**T**	**1,348**	**1.058-1.722**	**0,103**	**0,133**	**611**	**710**	**0,016**
DAPL1	rs2271663	2	159661077	C	T	0,983	0.843-1.147	0,465	0,460	611	695	0,832
DAPL1	rs17810428	2	159661451	G	A	0,951	0.808-1.119	0,321	0,312	611	703	0,544
DAPL1	rs9869	2	159663599	T	C	0,912	0.782-1.063	0,444	0,419	608	706	0,240
DAPL1	rs6716178	2	159667328	G	A	0,894	0.757-1.056	0,327	0,302	608	707	0,187
DAPL1	rs10016	2	159672442	A	G	0,937	0.800-1.096	0,370	0,353	610	706	0,414
DAPL1	rs11673825	2	159672626	T	C	0,921	0.782-1.085	0,300	0,284	609	707	0,327
MPP4	rs3754932	2	202509814	G	A	1,064	0.913-1.240	0,488	0,502	608	708	0,430
MPP4	rs2597900	2	202523347	G	A	1,036	0.888-1.208	0,428	0,437	608	702	0,655
MPP4	rs1208083	2	202532447	C	T	1,006	0.837-1.211	0,223	0,224	611	708	0,947
MPP4	rs1914267	2	202542836	G	A	0,954	0.816-1.115	0,435	0,424	607	707	0,554
MPP4	rs888012	2	202561571	C	T	0,958	0.808-1.137	0,294	0,286	608	704	0,625
RBP1	rs211585	3	139235564	G	A	1,021	0.876-1.189	0,476	0,481	610	708	0,792
RBP1	rs2071388	3	139236683	A	G	0,947	0.808-1.109	0,390	0,378	604	708	0,499
RBP1	rs10935331	3	139256732	G	C	1,062	0.911-1.237	0,416	0,430	611	708	0,444

RBP1	rs9862672	3	139262793	G	A	1,045	0.875-1.249	0,236	0,244	609	707	0,625
SUCNR1	rs6763405	3	151589418	G	A	1,034	0.775-1.382	0,076	0,079	594	699	0,821
SUCNR1	rs1402012	3	151589783	T	C	0,949	0.763-1.181	0,151	0,146	598	694	0,639
SUCNR1	rs1445359	3	151591741	T	C	1,261	0.985-1.618	0,102	0,124	596	691	0,067
SUCNR1	rs1445358	3	151593218	C	T	1,105	0.897-1.365	0,155	0,169	590	680	0,350
SUCNR1	rs13315275	3	151597310	G	A	1,031	0.877-1.212	0,418	0,426	596	684	0,712
SUCNR1	rs13079080	3	151599393	C	T	1,133	0.972-1.321	0,461	0,494	590	681	0,111
WDR17	rs17062505	4	177019413	T	C	0,961	0.814-1.136	0,307	0,300	610	704	0,643
WDR17	rs17625943	4	177098285	G	A	0,952	0.792-1.144	0,254	0,245	601	678	0,599
WDR17	rs11736872	4	177100644	G	A	0,924	0.782-1.092	0,292	0,276	610	708	0,356
NPVF	rs739749	7	25262594	A	G	1,026	0.880-1.196	0,482	0,489	601	689	0,743
NPVF	rs2074423	7	25263948	C	T	1,149	0.945-1.400	0,182	0,203	601	688	0,164
**RP1**	**rs9643828**	**8**	**55529073**	**T**	**C**	**0,835**	**0.704-0.989**	**0,295**	**0,260**	**612**	**710**	**0,037**
RP1	rs2293869	8	55539395	A	T	1,060	0.91-1.2340	0,426	0,439	605	702	0,458
RP1	rs446227	8	55541450	G	A	1,133	0.949-1.354	0,248	0,272	612	710	0,168
TRPM3	rs1889915	9	73164712	G	A	1,069	0.916-1.247	0,474	0,489	609	708	0,398
TRPM3	rs11142497	9	73198353	G	A	0,953	0.815-1.116	0,443	0,431	609	709	0,552
TRPM3	rs11142503	9	73218892	G	A	0,923	0.784-1.087	0,350	0,333	611	707	0,336
TRPM3	rs1538670	9	73255337	G	T	0,953	0.817-1.111	0,421	0,409	607	704	0,538
TRPM3	rs10123161	9	73296400	G	A	0,966	0.822-1.135	0,328	0,320	612	710	0,670
TRPM3	rs7031754	9	73311986	T	C	0,902	0.772-1.054	0,442	0,417	611	707	0,194
TRPM3	rs564929	9	73434585	G	A	1,079	0.922-1.263	0,361	0,378	612	707	0,342
TRPM3	rs579587	9	73438011	G	A	0,910	0.779-1.064	0,439	0,416	612	708	0,237
TRPM3	rs1337029	9	73459960	T	C	1,050	0.886-1.246	0,263	0,272	611	707	0,573
TRPM3	rs2152757	9	73478555	G	A	0,868	0.719-1.048	0,213	0,190	609	707	0,141
CDHR1	rs7099098	10	85950406	C	T	0,959	0.815-1.129	0,346	0,337	612	701	0,615
CDHR1	rs11200915	10	85957681	A	C	0,994	0.839-1.177	0,295	0,292	607	706	0,939
CDHR1	rs11200920	10	85960274	A	C	1,092	0.908-1.315	0,224	0,238	612	710	0,350
CDHR1	rs4933975	10	85960395	G	C	0,955	0.819-1.113	0,467	0,454	602	706	0,553
CDHR1	rs4933978	10	85971347	G	A	0,893	0.752-1.060	0,281	0,257	612	703	0,197
CDHR1	rs3814213	10	85974236	C	T	1,052	0.903-1.225	0,471	0,485	612	702	0,518
RGR	rs1042454	10	86012713	C	T	1,054	0.896-1.240	0,345	0,360	612	710	0,524
RGR	rs11200947	10	86020637	A	G	1,179	0.972-1.432	0,193	0,220	611	710	0,096
CTSD	rs2334411	11	1773477	A	C	0,944	0.789-1.129	0,260	0,247	603	699	0,528
CTSD	rs8839	11	1774136	A	C	1,071	0.867-1.324	0,152	0,161	610	706	0,528
CTSD	rs55923455	11	1777866	A	G	0,955	0.807-1.130	0,325	0,315	599	696	0,593
CTSD	rs1317356	11	1779138	G	A	1,076	0.921-1.257	0,489	0,508	609	707	0,355
CTSD	rs7122341	11	1781790	A	G	0,949	0.792-1.136	0,240	0,229	608	709	0,568
FADS3	rs174626	11	61637057	T	C	1,054	0.907-1.224	0,453	0,466	604	710	0,496
FADS3	rs174634	11	61647387	G	C	1,044	0.873-1.249	0,243	0,251	612	710	0,638
FADS3	rs174468	11	61663691	C	T	0,969	0.828-1.134	0,412	0,405	611	707	0,697
BEST1	rs149698	11	61730036	G	A	0,936	0.795-1.101	0,317	0,302	609	704	0,424
BEST1	rs1800008	11	61730183	C	T	1,105	0.920-1.328	0,234	0,250	610	705	0,288
BEST1	rs1800009	11	61730234	T	C	1,163	0.988-1.371	0,334	0,366	609	707	0,070
C11orf48	rs7386	11	62430335	C	T	0,908	0.782-1.053	0,482	0,455	611	708	0,201
C11orf48	rs17637597	11	62438750	A	G	1,135	0.956-1.348	0,257	0,283	611	709	0,149
RDH12	rs718212	14	68196636	T	C	0,910	0.776-1.067	0,400	0,381	608	704	0,247
RLBP1	rs2710	15	89753220	G	A	1,021	0.874-1.193	0,399	0,403	602	703	0,791
RLBP1	rs3825991	15	89761664	G	T	1,055	0.908-1.225	0,489	0,503	601	701	0,488
ATP1B2	rs1642764	17	7557834	T	C	1,032	0.884-1.205	0,456	0,462	608	707	0,688
ATP1B2	rs55831773	17	7559037	C	T	1,132	0.932-1.375	0,182	0,201	611	708	0,212
NETO1	rs11872857	18	70416119	A	G	1,020	0.809-1.288	0,119	0,122	611	710	0,868
NETO1	rs2000809	18	70435137	T	C	1,063	0.892-1.266	0,254	0,265	601	684	0,496
NETO1	rs1032102	18	70453405	G	A	1,035	0.876-1.224	0,299	0,305	612	704	0,683
NETO1	rs753147	18	70461059	G	A	0,912	0.779-1.069	0,425	0,405	612	708	0,256

NETO1	rs753744	18	70527649	G	A	0,915	0.766-1.094	0,274	0,256	612	708	0,331
NETO1	rs10164255	18	70532840	C	A	1,089	0.933-1.271	0,400	0,423	611	710	0,282
**CST3**	**rs2424577**	**20**	**23613750**	**C**	**T**	**1,246**	**1.025-1.517**	**0,181**	**0,215**	**611**	**707**	**0,028**
CST3	rs3787499	20	23616807	T	C	1,153	0.983-1.354	0,377	0,410	612	710	0,081

*from logistic regression model adjusted for age and gender

Supplementary Table S3. Association results for 524 imputed SNPs in discovery (710 late stage AMD/612 controls).

SNP (dbSNP-ID)	OR	95% CI	P-Value*	Minor Allele		Minor allele	Position [hg19]	Imputation quality
				Cases	Controls			
rs150838996	1,0145	0.356-2.969	0,978	0,008	0,008	C	158653244	0,692
rs145137393	1,0302	0.362-3.015	0,955	0,007	0,006	G	158668404	0,827
rs150622538	1,0329	0.364-3.023	0,951	0,007	0,006	C	158681719	0,835
rs139227362	1,0324	0.369-2.976	0,952	0,007	0,006	A	158754744	0,848
rs145406351	0,6741	0.205-2.133	0,500	0,007	0,009	C	158760865	0,575
rs140636113	1,0327	0.372-2.954	0,951	0,007	0,006	A	158768496	0,855
rs114391742	1,0516	0.382-2.982	0,922	0,010	0,010	A	158776246	0,568
rs115784579	1,0329	0.377-2.906	0,950	0,012	0,012	A	158778851	0,501
rs142646978	1,0322	0.374-2.93	0,951	0,007	0,006	T	158789944	0,863
rs141691508	1,0326	0.375-2.927	0,950	0,007	0,006	G	158800369	0,864
rs115892938	1,0562	0.383-3.004	0,916	0,009	0,009	A	158800615	0,638
rs146892138	1,0328	0.375-2.925	0,950	0,007	0,006	T	158802504	0,865
rs115181132	1,0278	0.374-2.908	0,958	0,008	0,008	A	158810887	0,712
rs139450184	1,0323	0.375-2.922	0,951	0,007	0,007	T	158819713	0,865
rs184003772	1,0149	0.369-2.867	0,977	0,008	0,008	A	158838065	0,745
rs116576403	1,0152	0.369-2.864	0,977	0,009	0,009	G	158850745	0,639
rs115790766	1,0152	0.369-2.864	0,977	0,009	0,009	G	158852586	0,639
rs115897593	1,0152	0.37-2.864	0,977	0,009	0,009	A	158853170	0,639
rs144305649	1,0153	0.37-2.864	0,976	0,009	0,009	T	158855768	0,639
rs115819307	1,0155	0.37-2.865	0,976	0,009	0,009	A	158856625	0,639
rs190633632	0,9999	0.364-2.813	1,000	0,009	0,008	A	158862789	0,681
rs183827630	0,9999	0.364-2.813	1,000	0,009	0,008	T	158862904	0,681
rs149905133	1,0328	0.377-2.915	0,950	0,007	0,007	C	158863278	0,868
rs116525992	1	0.364-2.813	1,000	0,009	0,008	C	158866864	0,681
rs139244229	0,9998	0.364-2.812	1,000	0,009	0,008	A	158873002	0,682
rs142220664	0,9948	0.363-2.794	0,992	0,009	0,009	T	158880224	0,658
rs116817954	0,9944	0.363-2.792	0,991	0,009	0,009	T	158880834	0,658
rs7577178	0,9946	0.363-2.792	0,992	0,009	0,009	T	158881129	0,659
rs7589538	0,9943	0.363-2.791	0,991	0,009	0,009	A	158881409	0,659
rs144649088	1,0595	0.393-2.946	0,909	0,010	0,010	C	158883799	0,573
rs137883435	0,9909	0.363-2.767	0,986	0,010	0,010	G	158887504	0,589
rs138218569	1,0318	0.384-2.851	0,950	0,007	0,007	G	158908471	0,888
rs150285152	1,0315	0.384-2.849	0,951	0,007	0,007	A	158917015	0,889
rs184761554	1,0319	0.384-2.849	0,950	0,007	0,007	T	158925598	0,889
rs142908507	1,0316	0.384-2.848	0,951	0,007	0,007	T	158928217	0,889
rs143975395	1,0216	0.386-2.772	0,966	0,011	0,010	T	158935158	0,581
rs142966580	1,0313	0.385-2.844	0,951	0,007	0,007	G	158967115	0,89
rs141322905	1,0311	0.385-2.837	0,951	0,007	0,007	C	159001591	0,892
rs138916318	1,0315	0.386-2.836	0,951	0,007	0,007	G	159018235	0,893
rs138602382	1,0277	0.389-2.79	0,956	0,008	0,008	G	159038216	0,757
rs112603366	1,0281	0.39-2.787	0,955	0,008	0,008	T	159052510	0,788
rs149810774	1,0492	0.399-2.842	0,922	0,008	0,007	C	159079891	0,863
rs141881062	1,0492	0.399-2.842	0,922	0,008	0,007	T	159081205	0,863

rs146258368	1,0592	0.405-2.856	0,907	0,008	0,007	T	159119833	0,83
rs141599227	1,0593	0.405-2.856	0,906	0,008	0,007	A	159134831	0,831
rs192047699	1,0328	0.394-2.781	0,948	0,010	0,010	T	159135175	0,643
rs143568364	1,059	0.405-2.855	0,907	0,008	0,007	T	159148086	0,831
rs151260863	1,059	0.405-2.855	0,907	0,008	0,007	C	159149624	0,831
rs145117131	1,059	0.405-2.855	0,907	0,008	0,007	C	159150119	0,831
rs145857501	1,0625	0.407-2.864	0,902	0,008	0,007	T	159150625	0,85
rs144201019	1,0588	0.405-2.854	0,907	0,008	0,007	A	159159973	0,831
rs139324464	1,0589	0.405-2.854	0,907	0,008	0,007	A	159162549	0,831
rs150030871	1,0589	0.405-2.854	0,907	0,008	0,007	A	159162562	0,831
rs138142378	1,0589	0.405-2.854	0,907	0,008	0,007	T	159166314	0,831
rs146695856	1,0589	0.405-2.854	0,907	0,008	0,007	G	159168216	0,831
rs144022306	1,0344	0.396-2.779	0,945	0,007	0,007	T	159178228	0,898
rs188231192	1,0158	0.389-2.719	0,974	0,008	0,008	T	159185564	0,784
rs180801045	1,0519	0.403-2.831	0,918	0,008	0,007	T	159187942	0,873
rs146315684	1,0347	0.396-2.779	0,944	0,007	0,007	G	159193740	0,898
rs113022182	1,0156	0.389-2.718	0,975	0,008	0,008	C	159247233	0,785
rs139900955	1,0415	0.406-2.748	0,933	0,011	0,010	A	159252478	0,629
rs144432246	1,0158	0.39-2.716	0,974	0,008	0,008	A	159258179	0,785
rs113829131	1,0159	0.39-2.717	0,974	0,008	0,008	A	159261524	0,785
rs138103149	1,0385	0.404-2.746	0,937	0,010	0,009	G	159269511	0,676
rs113076635	1,0419	0.406-2.749	0,932	0,011	0,010	T	159274998	0,629
rs143949519	1,0156	0.39-2.715	0,975	0,008	0,008	T	159279525	0,785
rs191093167	1,0134	0.389-2.709	0,978	0,008	0,008	T	159279966	0,784
rs112503256	1,0422	0.406-2.75	0,931	0,011	0,010	T	159283550	0,611
rs184838285	1,0153	0.39-2.714	0,975	0,008	0,008	G	159294640	0,785
rs113720556	1,0406	0.405-2.747	0,934	0,011	0,011	A	159298280	0,601
rs113276961	1,042	0.406-2.75	0,932	0,011	0,010	G	159298641	0,611
rs112096231	1,0154	0.39-2.714	0,975	0,008	0,008	C	159299751	0,786
rs140498443	1,037	0.399-2.774	0,941	0,007	0,007	A	159367519	0,909
rs80197176	1,0206	0.399-2.676	0,966	0,012	0,011	G	159448708	0,575
rs80100271	1,0209	0.399-2.676	0,965	0,012	0,011	A	159473103	0,576
rs190311626	0,7007	0.233-2.037	0,511	0,006	0,008	A	159510972	0,719
rs144179854	1,0413	0.401-2.781	0,934	0,009	0,008	A	159522161	0,782
rs142513824	1,0411	0.401-2.781	0,934	0,009	0,008	A	159527069	0,782
rs142334606	1,0412	0.401-2.781	0,934	0,009	0,008	A	159529043	0,782
rs114015675	1,0014	0.387-2.65	0,998	0,009	0,009	G	159550093	0,715
rs139703866	1,0014	0.387-2.65	0,998	0,009	0,009	A	159554500	0,715
rs116136901	1,0015	0.387-2.65	0,998	0,009	0,009	T	159556244	0,715
rs76287673	1,0015	0.387-2.65	0,998	0,009	0,009	G	159556928	0,715
rs116539093	1,0015	0.387-2.65	0,998	0,009	0,009	A	159557388	0,715
rs77770834	0,9759	0.382-2.539	0,959	0,011	0,011	T	159560918	0,609
rs146933283	0,9759	0.382-2.539	0,959	0,011	0,011	T	159561896	0,609
rs115928048	0,9763	0.382-2.54	0,960	0,011	0,011	T	159563768	0,609
rs113608782	1,04	0.406-2.736	0,935	0,010	0,010	A	159571403	0,645
rs112175045	1,0428	0.412-2.71	0,929	0,012	0,011	A	159583414	0,617
rs145597752	1,0188	0.398-2.676	0,969	0,009	0,008	G	159585797	0,793
rs143380470	1,0345	0.403-2.727	0,944	0,008	0,007	T	159591213	0,902
rs4665002	0,9887	0.781-1.252	0,925	0,233	0,232	C	159591798	0,545
rs145812200	0,9693	0.39-2.449	0,946	0,010	0,011	A	159593822	0,701

rs4665003	0,9859	0.801-1.214	0,894	0,328	0,328	T	159596315	0,56
rs58111038	0,9766	0.796-1.198	0,820	0,263	0,265	C	159596588	0,663
rs13423216	0,98	0.797-1.205	0,848	0,314	0,315	A	159596975	0,577
rs10186039	0,9617	0.781-1.184	0,713	0,319	0,323	T	159598589	0,565
rs55787037	0,9646	0.779-1.194	0,740	0,341	0,344	G	159598763	0,539
rs1996274	0,9615	0.781-1.184	0,711	0,319	0,323	T	159599779	0,565
rs115214475	0,9569	0.388-2.393	0,923	0,011	0,011	T	159599946	0,671
rs114892276	0,9569	0.388-2.393	0,923	0,011	0,011	C	159600411	0,671
rs141846279	0,957	0.388-2.392	0,923	0,011	0,011	C	159600734	0,671
rs4664263	0,962	0.781-1.186	0,716	0,319	0,323	A	159601129	0,56
rs62183011	0,9679	0.787-1.19	0,757	0,275	0,278	G	159601559	0,629
rs114663476	0,9573	0.389-2.391	0,924	0,011	0,011	C	159605376	0,672
rs1040166	0,8474	0.668-1.074	0,172	0,690	0,706	T	159606437	0,507
rs4362514	0,9428	0.787-1.13	0,523	0,282	0,290	A	159606976	0,814
rs7576266	0,9179	0.756-1.114	0,386	0,329	0,344	C	159607003	0,577
rs62183012	0,9426	0.786-1.13	0,522	0,282	0,290	C	159607760	0,814
rs62183013	0,9418	0.786-1.128	0,515	0,281	0,290	A	159607874	0,818
rs79029713	1,4177	1.058-1.909	0,020	0,144	0,120	T	159608659	0,609
rs60324897	1,4079	1.054-1.89	0,021	0,148	0,125	A	159608966	0,606
rs10497200	1,408	1.054-1.89	0,021	0,148	0,125	A	159610120	0,606
rs11681670	0,9445	0.79-1.129	0,529	0,271	0,279	A	159610292	0,86
rs142646819	1,4025	1.051-1.88	0,022	0,147	0,123	C	159611690	0,617
rs4508554	0,9206	0.758-1.119	0,405	0,318	0,332	T	159612431	0,598
rs76502338	1,407	1.054-1.887	0,021	0,148	0,124	A	159612450	0,611
rs6437198	0,9173	0.758-1.111	0,376	0,371	0,386	C	159613196	0,571
rs11678112	0,9479	0.796-1.129	0,548	0,279	0,287	A	159613337	0,879
rs76276633	1,4136	1.058-1.899	0,020	0,144	0,120	T	159613499	0,617
rs17203501	0,9474	0.796-1.128	0,543	0,280	0,288	A	159613637	0,883
rs10451596	0,9121	0.754-1.103	0,343	0,366	0,382	C	159613661	0,58
rs10497201	0,9474	0.796-1.128	0,543	0,280	0,288	C	159614144	0,883
rs6733595	0,9108	0.752-1.103	0,338	0,360	0,376	C	159614497	0,573
rs200382940	0,9459	0.794-1.127	0,534	0,280	0,289	C	159615537	0,875
rs62183014	0,947	0.794-1.13	0,545	0,277	0,285	T	159615541	0,868
rs1515921	0,9473	0.796-1.128	0,542	0,280	0,288	G	159615668	0,883
rs75962772	1,4073	1.054-1.887	0,021	0,148	0,124	G	159615755	0,61
rs1515920	0,9483	0.797-1.129	0,550	0,281	0,289	C	159615764	0,88
rs35026117	0,9106	0.752-1.103	0,339	0,362	0,378	AT	159616018	0,57
rs10497202	1,407	1.054-1.887	0,021	0,148	0,124	C	159616934	0,611
rs80335441	1,4071	1.054-1.887	0,021	0,148	0,124	A	159617834	0,611
rs72993498	0,9479	0.399-2.279	0,903	0,015	0,015	T	159618104	0,534
rs60864902	1,4101	1.055-1.894	0,021	0,147	0,123	C	159618280	0,614
rs72937273	0,9473	0.796-1.128	0,542	0,280	0,288	G	159618716	0,883
rs56894726	1,4054	1.052-1.886	0,022	0,145	0,121	C	159619146	0,625
rs11683568	0,9463	0.795-1.127	0,536	0,285	0,294	C	159619202	0,866
rs139858290	1,406	1.052-1.887	0,022	0,144	0,121	A	159619820	0,625
rs11422552	1,3543	1.016-1.812	0,040	0,183	0,162	TA	159620245	0,519
rs62183016	0,9476	0.797-1.127	0,542	0,281	0,290	C	159620530	0,89
rs10933502	0,9196	0.76-1.112	0,388	0,344	0,359	C	159621237	0,588
rs80155867	1,1472	0.12-13.04	0,904	0,002	0,002	A	159621384	0,505
rs148057425	1,4059	1.052-1.887	0,022	0,144	0,121	A	159621470	0,625

rs77174026	0,9515	0.4-2.288	0,910	0,015	0,015	A	159621715	0,54
rs76719473	1,1472	0.12-13.04	0,904	0,002	0,002	C	159621788	0,505
rs76686128	1,4056	1.052-1.886	0,022	0,145	0,121	A	159622055	0,626
rs1515926	0,9202	0.758-1.117	0,400	0,324	0,338	C	159622208	0,597
rs189976173	0,7469	0.256-2.133	0,582	0,006	0,007	A	159622444	0,832
rs80336821	1,1472	0.12-13.04	0,904	0,002	0,002	C	159622625	0,505
rs62183017	0,9476	0.797-1.126	0,541	0,282	0,291	A	159622852	0,894
rs145657727	1,4164	1.051-1.917	0,023	0,141	0,118	T	159623085	0,604
rs6730860	0,9207	0.758-1.118	0,403	0,324	0,338	T	159623524	0,596
rs10933503	0,9209	0.762-1.113	0,394	0,348	0,362	T	159624015	0,588
rs55989586	1,3745	1.031-1.84	0,031	0,158	0,136	C	159624664	0,581
rs60731215	1,0704	0.902-1.271	0,436	0,428	0,413	A	159624706	0,743
rs140624122	1,1472	0.12-13.04	0,904	0,002	0,002	T	159625166	0,505
rs141866937	0,9476	0.797-1.126	0,541	0,282	0,291	A	159625476	0,894
rs1913901	1,0717	0.903-1.273	0,429	0,429	0,413	A	159625533	0,74
rs16843331	1,4102	1.056-1.892	0,021	0,146	0,122	G	159625777	0,621
rs1515928	1,0705	0.902-1.271	0,436	0,428	0,413	G	159625983	0,743
rs80284529	1,4092	1.055-1.892	0,021	0,142	0,118	T	159625985	0,633
rs72937289	0,9477	0.798-1.126	0,541	0,282	0,291	A	159627512	0,894
rs62183019	0,9477	0.798-1.126	0,541	0,282	0,291	A	159628366	0,894
rs77664069	1,4014	1.05-1.878	0,023	0,142	0,118	T	159628595	0,643
rs76719581	1,4065	1.054-1.886	0,022	0,144	0,120	T	159628604	0,633
rs72995111	0,9814	0.412-2.37	0,966	0,012	0,013	G	159628881	0,649
rs77270274	1,1472	0.12-13.04	0,904	0,002	0,002	G	159628896	0,506
rs4665005	1,0549	0.875-1.271	0,574	0,546	0,539	G	159629418	0,606
rs1515929	0,9514	0.803-1.127	0,564	0,281	0,289	G	159629637	0,929
rs12611652	1,0533	0.879-1.263	0,575	0,531	0,523	A	159629994	0,636
rs11689479	1,0514	0.878-1.259	0,586	0,527	0,520	A	159630705	0,647
rs79632155	1,3612	1.026-1.813	0,033	0,139	0,116	A	159630744	0,679
rs17809948	0,9537	0.808-1.125	0,574	0,288	0,296	T	159633415	0,964
rs72937298	0,9536	0.808-1.125	0,573	0,288	0,296	C	159636048	0,964
rs13415114	1,0528	0.88-1.26	0,574	0,524	0,516	A	159636686	0,642
rs76721474	1,4	1.053-1.871	0,022	0,112	0,088	C	159638393	0,818
rs9967675	0,9239	0.772-1.106	0,388	0,339	0,354	C	159638587	0,686
rs77227830	1,1782	0.123-13.503	0,886	0,002	0,002	G	159638741	0,588
rs62183660	0,9606	0.813-1.136	0,638	0,298	0,304	G	159639316	0,927
rs2356507	0,9224	0.77-1.104	0,379	0,350	0,366	T	159639345	0,658
rs62183661	0,9539	0.809-1.125	0,575	0,287	0,296	A	159639718	0,967
rs79614044	1,319	1.019-1.713	0,036	0,124	0,100	A	159641297	0,91
rs201857133	0,919	0.767-1.101	0,361	0,325	0,342	TA	159641436	0,706
rs112392497	1,009	0.423-2.452	0,984	0,013	0,013	T	159641443	0,616
rs2138387	0,915	0.763-1.097	0,338	0,333	0,350	A	159641444	0,686
rs144798268	1,1372	0.538-2.45	0,738	0,017	0,015	C	159641554	0,664
rs62183662	0,9471	0.8-1.121	0,528	0,316	0,325	G	159641579	0,879
rs72939206	0,9476	0.801-1.122	0,532	0,313	0,322	A	159642311	0,883
rs181677566	1,1513	0.545-2.48	0,714	0,016	0,014	C	159642531	0,694
rs72939209	0,9471	0.8-1.121	0,527	0,313	0,323	G	159642803	0,883
rs142844306	0,9478	0.801-1.122	0,532	0,311	0,320	GGA	159643052	0,888
rs147441846	1,3181	1.019-1.712	0,037	0,123	0,099	A	159643141	0,918
rs4027725	1,0461	0.727-1.503	0,808	0,900	0,898	C	159643173	0,529

rs57214770	0,947	0.8-1.121	0,527	0,313	0,323	T	159643229	0,883
rs76223778	1,1782	0.123-13.503	0,886	0,002	0,002	A	159643484	0,589
rs79281718	1,3181	1.019-1.712	0,037	0,123	0,099	A	159643742	0,918
rs2356508	1,0058	0.694-1.454	0,976	0,912	0,911	C	159644006	0,569
rs11691004	0,9573	0.812-1.129	0,604	0,287	0,294	T	159644175	0,972
rs17204323	1,3181	1.019-1.712	0,037	0,123	0,099	G	159645078	0,918
rs4254463	1,0348	0.876-1.223	0,688	0,481	0,475	G	159645941	0,784
rs62183665	0,9591	0.81-1.135	0,627	0,282	0,289	A	159646375	0,939
rs143577322	1,1782	0.123-13.503	0,886	0,002	0,002	T	159646846	0,59
rs72995137	0,9645	0.404-2.335	0,935	0,011	0,012	T	159647113	0,695
rs62183666	1,0675	0.919-1.24	0,393	0,420	0,401	A	159647191	0,977
rs111798763	1,3616	0.748-2.512	0,316	0,032	0,027	A	159648279	0,563
rs57672539	0,9483	0.8-1.125	0,542	0,306	0,315	C	159648393	0,877
rs144818987	0,9481	0.799-1.125	0,540	0,307	0,315	C	159648401	0,877
rs148268005	1,348	1.039-1.757	0,026	0,127	0,102	C	159648405	0,875
rs6729542	1,3507	1.04-1.762	0,025	0,125	0,099	C	159648421	0,885
rs146385127	0,9991	0.144-7.594	0,999	0,002	0,002	GCCA	159648853	0,666
rs1515924	0,9574	0.813-1.127	0,601	0,289	0,296	T	159649340	0,985
rs77003615	0,9991	0.144-7.594	0,999	0,002	0,002	G	159649434	0,666
rs7580547	0,8977	0.756-1.065	0,217	0,292	0,315	T	159649545	0,833
rs10933505	0,9568	0.821-1.114	0,570	0,463	0,475	A	159650308	0,981
rs4561615	1,2987	1.021-1.658	0,035	0,136	0,109	C	159650386	0,963
rs13401347	1,0668	0.777-1.466	0,689	0,126	0,122	A	159650855	0,588
rs77099722	0,9991	0.144-7.594	0,999	0,002	0,002	C	159650881	0,666
rs925781	1,067	0.913-1.248	0,416	0,600	0,586	G	159651734	0,993
rs1356173	0,9572	0.813-1.127	0,600	0,289	0,297	A	159651973	0,984
rs2280184	1,0785	0.923-1.261	0,343	0,601	0,584	T	159652231	1
rs12463934	0,8977	0.756-1.066	0,217	0,292	0,315	T	159652852	0,833
rs62183690	0,9563	0.812-1.126	0,591	0,289	0,297	A	159654244	0,985
rs908404	0,8978	0.756-1.066	0,218	0,292	0,316	T	159654380	0,833
rs925780	1,0866	0.781-1.51	0,621	0,916	0,911	C	159655103	0,724
rs6437202	1,0694	0.915-1.25	0,399	0,601	0,586	G	159656114	0,996
rs6722518	0,8977	0.756-1.066	0,217	0,293	0,316	T	159656272	0,832
rs55962574	0,8572	0.364-2.026	0,722	0,012	0,013	A	159656531	0,653
rs115127277	1,0588	0.15-8.412	0,954	0,002	0,002	C	159656700	0,69
rs112805562	1,3715	0.753-2.531	0,305	0,032	0,027	A	159657885	0,565
rs111676703	1,3728	0.754-2.534	0,304	0,032	0,027	T	159658591	0,566
rs78765308	1,307	1.027-1.669	0,031	0,132	0,105	A	159659291	0,998
rs6756488	0,8982	0.757-1.066	0,219	0,293	0,316	A	159660277	0,834
rs75277023	1,3081	1.028-1.67	0,030	0,132	0,105	A	159660494	0,999
rs200987154	1,0873	0.782-1.51	0,617	0,916	0,911	A	159660637	0,726
rs11297084	1,0597	0.76-1.477	0,732	0,908	0,905	T	159660645	0,666
rs17810398	1,3135	1.033-1.676	0,027	0,133	0,105	T	159660870	1
rs2271663	0,9846	0.845-1.147	0,842	0,459	0,464	T	159661077	1
rs17810428	0,9366	0.796-1.102	0,430	0,308	0,321	A	159661451	1
rs3914570	0,9265	0.794-1.081	0,331	0,428	0,448	A	159661485	0,937
rs11679019	0,9576	0.811-1.131	0,609	0,284	0,291	T	159661511	0,947
rs115509295	1,3777	0.757-2.543	0,299	0,032	0,027	A	159661539	0,567
rs78959770	1,3501	0.805-2.287	0,258	0,043	0,036	A	159661731	0,574
esv2676504	1,3093	1.025-1.678	0,032	0,134	0,108	T	159661994	0,951

rs1878099	1,0331	0.883-1.209	0,685	0,579	0,573	G	159662905	0,931
rs9288722	1,0326	0.882-1.21	0,691	0,581	0,575	C	159663099	0,923
rs9288723	1,0313	0.881-1.208	0,702	0,580	0,575	G	159663202	0,923
rs34097395	1,0426	0.888-1.225	0,611	0,600	0,592	A	159663359	0,921
rs9869	0,9132	0.784-1.064	0,244	0,418	0,443	C	159663599	1
rs10497199	0,9627	0.819-1.132	0,645	0,292	0,298	A	159663616	0,965
rs11690492	1,073	0.749-1.539	0,701	0,097	0,094	C	159664171	0,581
rs62183691	0,9637	0.819-1.134	0,656	0,293	0,299	A	159664285	0,955
rs143039350	0,9763	0.408-2.369	0,957	0,011	0,011	A	159664435	0,704
rs17810584	1,294	1.009-1.666	0,044	0,132	0,108	T	159664703	0,918
rs10188387	1,2867	1.002-1.658	0,049	0,145	0,121	T	159664713	0,825
rs79362544	1,3874	0.773-2.523	0,276	0,035	0,029	C	159665095	0,551
rs79649315	1,2877	1.002-1.661	0,050	0,130	0,107	T	159665382	0,908
rs1980154	1,0727	0.735-1.563	0,715	0,931	0,927	C	159666072	0,648
rs4459689	0,9597	0.815-1.13	0,621	0,285	0,292	C	159666152	0,95
rs1356172	1,0371	0.711-1.51	0,849	0,923	0,922	G	159666619	0,604
rs1105824	0,955	0.814-1.121	0,574	0,290	0,298	A	159667098	0,911
rs908403	0,8302	0.663-1.038	0,104	0,782	0,803	T	159667297	0,734
rs6716178	0,9033	0.765-1.067	0,230	0,302	0,325	A	159667328	1
rs908402	1,0351	0.707-1.513	0,859	0,924	0,923	G	159667731	0,598
rs4665011	0,8436	0.693-1.026	0,089	0,643	0,669	C	159668758	0,753
rs2102749	1,0071	0.691-1.466	0,971	0,917	0,916	C	159669048	0,565
rs2090300	0,834	0.673-1.032	0,095	0,758	0,781	A	159669303	0,728
rs75409857	1,6733	0.184-22.05	0,655	0,002	0,001	T	159669561	0,672
rs6437203	0,8348	0.674-1.033	0,098	0,758	0,780	G	159669573	0,724
rs4665013	0,9317	0.785-1.106	0,418	0,313	0,329	G	159669797	0,882
rs114372196	1,7305	0.189-23.467	0,636	0,002	0,001	C	159670025	0,673
rs111634981	1,0984	0.796-1.519	0,568	0,085	0,080	A	159670137	0,765
rs13004210	1,0839	0.912-1.288	0,359	0,569	0,551	G	159670211	0,653
rs74923781	1,399	1.072-1.835	0,014	0,135	0,107	G	159670440	0,722
rs16843372	1,0497	0.889-1.24	0,567	0,605	0,593	C	159670449	0,749
rs11693126	0,8672	0.677-1.11	0,259	0,191	0,204	C	159670945	0,569
rs10169705	1,1519	0.967-1.374	0,114	0,352	0,326	T	159671744	0,906
rs17810816	1,3801	1.064-1.799	0,016	0,140	0,112	G	159671992	0,765
rs1515922	1,0253	0.793-1.326	0,849	0,161	0,160	A	159672168	0,669
rs61740880	1,3302	0.778-2.294	0,300	0,042	0,035	T	159672251	0,582
rs61740878	1,0106	0.777-1.316	0,938	0,146	0,146	A	159672252	0,674
rs10016	1,0659	0.911-1.247	0,424	0,646	0,630	A	159672442	1
rs11673825	0,9271	0.788-1.091	0,363	0,285	0,300	C	159672626	1
rs17810918	1,338	1.049-1.712	0,020	0,151	0,122	A	159674084	0,832
rs78599894	0,9194	0.781-1.083	0,313	0,277	0,294	A	159674424	0,989
rs10168849	1,1566	0.97-1.38	0,106	0,352	0,326	C	159674431	0,912
rs13386910	1,1774	0.977-1.421	0,088	0,329	0,303	C	159674622	0,829
rs13398448	1,1768	0.976-1.421	0,090	0,328	0,302	A	159674631	0,826
rs7603880	1,0431	0.891-1.221	0,599	0,645	0,634	T	159674805	0,948
rs72943123	0,9189	0.78-1.082	0,310	0,277	0,294	A	159675223	0,991
rs13390359	1,0531	0.895-1.239	0,532	0,610	0,597	C	159675307	0,856
rs74556943	0,9188	0.78-1.082	0,310	0,277	0,294	C	159675723	0,991
rs13393279	1,0431	0.891-1.221	0,599	0,645	0,634	C	159675728	0,948
rs10172746	1,0461	0.891-1.229	0,583	0,621	0,610	C	159676098	0,88

rs10207728	1,0478	0.89-1.233	0,574	0,656	0,645	T	159676116	0,929
rs72997107	1,319	1.033-1.69	0,027	0,171	0,144	T	159676285	0,741
rs61482440	1,3199	1.034-1.691	0,027	0,171	0,144	A	159676470	0,742
rs11694423	0,9155	0.774-1.083	0,302	0,273	0,289	A	159676531	0,951
rs28523325	1,047	0.889-1.233	0,582	0,615	0,604	G	159677351	0,853
rs11696015	0,8402	0.649-1.088	0,186	0,171	0,185	T	159677556	0,6
rs72997111	1,319	1.033-1.69	0,027	0,170	0,143	T	159677635	0,743
rs10167350	1,0095	0.779-1.31	0,943	0,150	0,150	G	159678360	0,683
rs10182297	1,0487	0.889-1.237	0,573	0,649	0,638	C	159678752	0,899
rs10184861	1,0487	0.889-1.237	0,573	0,649	0,638	A	159678761	0,899
rs72997116	1,0482	0.89-1.235	0,573	0,611	0,600	G	159679656	0,845
rs13416455	1,0459	0.888-1.232	0,591	0,608	0,597	A	159679672	0,841
rs13404977	1,0459	0.888-1.232	0,591	0,608	0,597	T	159679756	0,841
rs7595343	1,0871	0.857-1.378	0,490	0,789	0,779	T	159679785	0,581
rs13416676	1,1906	0.983-1.444	0,076	0,319	0,292	A	159679819	0,827
rs11688793	1,0482	0.89-1.235	0,573	0,611	0,600	A	159679866	0,845
rs10196949	1,04E+00	0.882-1.227	0,640	0,598	0,588	A	159680230	0,823
rs10206348	1,0098	0.774-1.318	0,942	0,147	0,147	T	159680730	0,664
rs10174267	1,0494	0.888-1.24	0,571	0,643	0,633	A	159681141	0,876
rs13392727	1,0115	0.778-1.316	0,932	0,148	0,148	A	159681180	0,672
rs5835723	1,0446	0.885-1.233	0,606	0,597	0,587	CT	159682761	0,817
rs11685379	0,9112	0.763-1.087	0,303	0,258	0,274	A	159683357	0,89
rs201101769	0,8768	0.669-1.148	0,339	0,162	0,172	GT	159683880	0,502
rs2138386	1,0429	0.884-1.231	0,618	0,600	0,590	T	159683886	0,823
rs35854390	1,0424	0.882-1.232	0,625	0,593	0,583	A	159684051	0,799
rs138937208	1,3631	0.759-2.468	0,302	0,038	0,033	T	159684153	0,529
rs77861063	1,6914	0.186-22.367	0,649	0,003	0,002	C	159684186	0,5
rs13403565	1,0406	0.882-1.228	0,637	0,597	0,587	C	159684204	0,82
rs35671724	1,1915	0.983-1.446	0,076	0,318	0,291	T	159684919	0,823
rs13410936	1,0467	0.886-1.237	0,591	0,585	0,575	C	159686370	0,801
rs62183695	1,0727	0.778-1.482	0,669	0,104	0,101	T	159686509	0,628
rs13397887	1,0448	0.885-1.234	0,605	0,590	0,580	T	159686659	0,809
rs76080809	1,6914	0.186-22.367	0,649	0,003	0,002	T	159687038	0,5
rs13423228	1,1922	0.983-1.447	0,074	0,318	0,291	A	159687255	0,823
rs13398569	1,0448	0.885-1.234	0,605	0,590	0,580	T	159687276	0,809
rs13398901	1,0448	0.885-1.234	0,605	0,590	0,580	T	159687570	0,809
rs6744604	1,0484	0.887-1.239	0,579	0,643	0,632	G	159687895	0,871
rs149174156	0,914	0.77-1.085	0,304	0,266	0,282	T	159688006	0,923
rs148363671	1,6914	0.186-22.367	0,649	0,003	0,002	A	159688121	0,5
rs115720768	1,6914	0.186-22.367	0,649	0,003	0,002	A	159688202	0,5
rs190899037	1,6914	0.186-22.367	0,649	0,003	0,002	T	159688784	0,5
rs12694970	1,0435	0.881-1.236	0,622	0,578	0,568	T	159688785	0,758
rs141103629	0,9117	0.768-1.082	0,291	0,273	0,290	T	159689527	0,905
rs2356510	1,0473	0.887-1.237	0,586	0,594	0,583	C	159689830	0,811
rs72997133	1,157	0.967-1.385	0,111	0,366	0,340	C	159690368	0,89
rs13425388	1,1919	0.982-1.448	0,076	0,317	0,290	G	159690387	0,818
rs13425522	1,1865	0.982-1.435	0,077	0,326	0,299	C	159690542	0,836
rs189337808	0,3312	0.083-1.208	0,101	0,005	0,009	A	159690926	0,566
rs67774385	1,1923	0.983-1.447	0,074	0,318	0,291	A	159691409	0,823
rs77923387	1,6914	0.186-22.367	0,649	0,003	0,002	A	159691846	0,5

rs11883861	1,1578	0.968-1.385	0,109	0,367	0,341	A	159693064	0,893
rs70994236	1,1554	0.971-1.376	0,104	0,379	0,352	T	159693154	0,927
rs34525522	1,0508	0.888-1.243	0,563	0,643	0,632	G	159693823	0,866
rs62183715	1,1581	0.969-1.386	0,108	0,368	0,341	T	159693830	0,895
rs147201788	1,1395	0.54-2.452	0,733	0,015	0,014	T	159693847	0,716
rs1400018	1,1586	0.969-1.387	0,108	0,369	0,343	A	159694081	0,888
rs77111010	1,6914	0.186-22.367	0,649	0,003	0,002	A	159694487	0,5
rs76399313	1,6914	0.186-22.367	0,649	0,003	0,002	T	159694565	0,5
rs67385905	1,0122	0.776-1.322	0,929	0,150	0,150	A	159694639	0,654
rs1807921	1,0466	0.882-1.242	0,602	0,622	0,613	C	159694749	0,816
rs10194836	1,0482	0.884-1.243	0,589	0,629	0,619	C	159694940	0,829
rs72997145	1,3273	1.034-1.71	0,027	0,170	0,143	T	159695064	0,717
rs10184397	1,0442	0.88-1.239	0,620	0,632	0,622	T	159695242	0,831
rs10184590	1,1867	0.979-1.439	0,081	0,326	0,299	T	159695384	0,824
rs57750082	1,1867	0.979-1.439	0,081	0,326	0,299	G	159695547	0,824
rs10190307	1,0503	0.885-1.247	0,575	0,633	0,623	C	159696188	0,821
rs10175732	1,0475	0.883-1.243	0,594	0,637	0,627	T	159696287	0,833
rs34063526	1,1858	0.977-1.44	0,084	0,322	0,296	G	159696659	0,818
rs56410323	0,9135	0.768-1.087	0,308	0,265	0,281	G	159696883	0,904
rs56174131	0,9062	0.755-1.088	0,291	0,323	0,338	G	159696889	0,747
rs10201455	1,1863	0.978-1.441	0,084	0,321	0,295	A	159696895	0,817
rs13416615	1,151	0.966-1.372	0,116	0,377	0,351	C	159697695	0,916
rs141143637	0,9135	0.768-1.087	0,308	0,265	0,281	C	159697721	0,904
rs11897100	1,1604	0.972-1.387	0,101	0,373	0,346	G	159698052	0,898
rs11898243	1,1585	0.971-1.384	0,104	0,375	0,348	C	159698059	0,902
rs140110959	1,1399	0.54-2.453	0,733	0,015	0,014	C	159699083	0,716
rs13421077	1,187	0.978-1.442	0,083	0,321	0,295	C	159699517	0,817
rs62183718	1,187	0.978-1.442	0,083	0,321	0,295	A	159699528	0,817
rs201231178	1,239	0.979-1.571	0,076	0,260	0,238	TC	159699689	0,626
rs78281143	1,2391	0.979-1.571	0,075	0,260	0,238	C	159699690	0,627
rs112501440	0,9135	0.768-1.087	0,307	0,265	0,281	G	159699902	0,904
rs12694971	1,187	0.978-1.442	0,083	0,321	0,295	T	159700075	0,817
rs72943174	0,9149	0.765-1.095	0,331	0,256	0,271	C	159700200	0,861
rs72943176	0,9149	0.765-1.095	0,331	0,256	0,271	T	159700201	0,861
rs72943178	0,9102	0.765-1.083	0,289	0,266	0,283	A	159700219	0,899
rs1125354	0,9135	0.768-1.087	0,307	0,265	0,281	A	159700565	0,904
rs1125355	0,9135	0.768-1.087	0,307	0,265	0,281	A	159700580	0,904
rs6709960	1,187	0.978-1.442	0,083	0,321	0,295	C	159700728	0,817
rs6754363	1,187	0.978-1.442	0,083	0,321	0,295	G	159700824	0,817
rs6754374	1,187	0.978-1.442	0,083	0,321	0,295	G	159700858	0,817
rs72939960	0,9135	0.768-1.087	0,308	0,265	0,281	A	159701160	0,904
rs13393182	1,1869	0.978-1.442	0,084	0,320	0,294	T	159701376	0,816
rs13417941	1,1869	0.978-1.442	0,084	0,320	0,294	A	159701386	0,816
rs13393248	1,0465	0.883-1.24	0,599	0,586	0,576	A	159701395	0,781
rs1403289	1,0463	0.883-1.239	0,600	0,586	0,576	T	159701572	0,781
rs72939969	0,9139	0.768-1.087	0,310	0,266	0,282	G	159701768	0,902
rs10204344	1,1864	0.977-1.441	0,084	0,321	0,295	A	159703397	0,817
rs150852266	0,9125	0.766-1.087	0,306	0,262	0,278	G	159704226	0,892
rs139338777	0,9124	0.766-1.087	0,305	0,263	0,279	T	159704293	0,891
rs13428776	1,0524	0.889-1.246	0,554	0,593	0,581	A	159704461	0,786

rs62183722	0,9121	0.764-1.089	0,308	0,283	0,298	C	159705113	0,829
rs10210845	1,0092	0.776-1.315	0,945	0,150	0,150	A	159705341	0,667
rs12694972	1,1869	0.978-1.442	0,083	0,321	0,295	C	159705609	0,817
rs12694973	1,0074	0.776-1.309	0,956	0,152	0,153	A	159705696	0,668
rs12694974	1,0496	0.886-1.244	0,576	0,645	0,634	C	159705870	0,849
rs12992591	1,187	0.978-1.442	0,083	0,321	0,295	T	159705988	0,817
rs114087196	0,9133	0.767-1.087	0,307	0,265	0,281	A	159706148	0,903
rs115049089	0,8283	0.627-1.093	0,184	0,159	0,173	A	159706162	0,535
rs116204562	0,9144	0.768-1.088	0,314	0,267	0,283	T	159706225	0,897
rs113194620	0,9133	0.767-1.087	0,307	0,265	0,281	A	159706469	0,903
rs61034713	1,0961	0.858-1.4	0,462	0,784	0,774	C	159706768	0,537
rs149884088	1,3406	1.043-1.729	0,023	0,153	0,126	G	159706790	0,774
rs60384835	1,0461	0.879-1.245	0,612	0,635	0,625	T	159706791	0,8
rs111609722	0,9131	0.766-1.088	0,308	0,267	0,283	G	159706845	0,889
rs114092151	0,9119	0.765-1.087	0,304	0,264	0,280	A	159706903	0,886
rs58176620	1,0128	0.794-1.293	0,918	0,187	0,186	C	159706983	0,635
rs7419557	1,013	0.803-1.279	0,913	0,210	0,210	G	159707062	0,647
rs7423023	1,1843	0.975-1.44	0,088	0,384	0,358	T	159707106	0,761
rs7421715	1,0622	0.882-1.28	0,525	0,639	0,628	C	159707125	0,689
rs56056398	1,0554	0.885-1.259	0,549	0,469	0,457	A	159707126	0,622
rs140343416	1,3348	1.026-1.742	0,032	0,201	0,176	A	159707144	0,542
rs7419566	1,192	0.977-1.456	0,084	0,320	0,295	G	159707152	0,775
rs6437204	1,1966	0.959-1.495	0,113	0,518	0,496	A	159707173	0,507
rs201054768	1,1064	0.905-1.353	0,324	0,549	0,534	TAC	159707190	0,588
rs150158829	1,3377	1.039-1.728	0,025	0,151	0,124	A	159707210	0,774
rs6437205	1,0663	0.894-1.271	0,474	0,635	0,622	A	159707236	0,781
rs6437206	1,0659	0.894-1.271	0,476	0,635	0,622	T	159707242	0,783
rs113361777	1,3377	1.039-1.728	0,025	0,151	0,124	T	159707249	0,774
rs56249648	0,9098	0.759-1.091	0,307	0,260	0,275	T	159707462	0,82
rs6437207	1,1	0.904-1.34	0,343	0,431	0,416	G	159707842	0,523
rs7562293	1,339	1.039-1.732	0,025	0,151	0,124	T	159707998	0,767
rs140328043	1,339	1.039-1.732	0,025	0,151	0,124	T	159708160	0,767
rs145072711	0,8721	0.683-1.114	0,273	0,200	0,213	T	159709151	0,573
rs149121711	1,33	1.038-1.709	0,025	0,176	0,149	C	159709248	0,651
rs151023383	0,8756	0.69-1.11	0,273	0,206	0,219	A	159709382	0,592
rs7592231	0,8546	0.67-1.09	0,205	0,233	0,247	C	159709452	0,517
rs56121680	0,8756	0.69-1.11	0,273	0,206	0,219	A	159709626	0,592
rs56061100	0,8756	0.69-1.11	0,273	0,206	0,219	A	159709682	0,592
rs113131637	1,3416	1.042-1.733	0,023	0,159	0,131	T	159710656	0,732
rs139792633	1,3458	0.775-2.355	0,294	0,042	0,035	C	159710740	0,548
rs186822967	0,8755	0.69-1.11	0,273	0,206	0,219	T	159710943	0,591
rs59737258	1,3398	1.05-1.716	0,019	0,183	0,154	A	159710981	0,717
rs111494772	1,34	1.039-1.735	0,025	0,152	0,125	T	159711036	0,757
rs72939987	0,8756	0.69-1.11	0,273	0,206	0,219	T	159711766	0,591
rs7583501	1,0067	0.763-1.329	0,963	0,145	0,146	A	159712283	0,623
rs6737129	1,338	1.04-1.728	0,025	0,152	0,125	A	159712710	0,769
rs150262494	0,8756	0.69-1.11	0,273	0,206	0,219	A	159712773	0,591
rs137932554	0,8756	0.69-1.11	0,273	0,206	0,219	C	159712995	0,591
rs148654825	0,8752	0.689-1.111	0,273	0,205	0,218	C	159713149	0,589
rs111286560	0,8756	0.69-1.11	0,273	0,206	0,219	A	159714582	0,591

rs112091414	0,8756	0.69-1.11	0,273	0,206	0,219	A	159714588	0,591
rs113957777	0,8752	0.689-1.111	0,273	0,205	0,218	A	159715018	0,589
rs113432711	0,8756	0.69-1.11	0,273	0,206	0,219	G	159715359	0,591
rs145271736	0,8756	0.69-1.11	0,273	0,206	0,219	C	159715793	0,591
rs139700769	1,3379	1.04-1.728	0,025	0,152	0,125	T	159716306	0,769
rs149026751	0,8756	0.69-1.11	0,273	0,206	0,219	T	159716425	0,591
rs115239849	0,8756	0.69-1.11	0,273	0,206	0,219	C	159716470	0,591
rs72939991	0,8756	0.69-1.11	0,273	0,206	0,219	T	159716557	0,591
rs149558988	0,8761	0.691-1.11	0,273	0,207	0,220	G	159716764	0,594
rs112677094	0,8539	0.668-1.09	0,205	0,240	0,255	G	159717420	0,503
rs60547757	1,0975	0.899-1.341	0,361	0,425	0,410	G	159717545	0,509
rs146948267	1,339	1.013-1.777	0,041	0,122	0,100	A	159717629	0,755
rs142435504	0,8752	0.689-1.111	0,273	0,205	0,218	G	159718589	0,589
rs13400326	1,0075	0.764-1.33	0,958	0,145	0,145	G	159718593	0,628
rs144087548	1,3393	1.04-1.732	0,025	0,151	0,125	T	159718894	0,765
rs145953089	0,8752	0.689-1.111	0,273	0,205	0,218	C	159719127	0,589
rs113383584	1,338	1.04-1.728	0,025	0,152	0,125	G	159719907	0,769
rs114739798	1,055	0.419-2.731	0,909	0,012	0,011	T	159719909	0,599
rs9751561	1,099	0.902-1.339	0,348	0,433	0,418	C	159719958	0,521
rs115455608	0,8756	0.69-1.11	0,273	0,206	0,219	T	159720289	0,591
rs9751590	1,3172	1.022-1.703	0,034	0,180	0,155	C	159720291	0,664
rs9751607	1,0923	0.9-1.325	0,371	0,592	0,577	C	159720488	0,518
rs55761371	1,0766	0.886-1.309	0,459	0,392	0,380	G	159720950	0,529
rs113340479	1,3379	1.04-1.728	0,025	0,152	0,125	T	159721099	0,769
rs71406131	1,0077	0.764-1.33	0,957	0,145	0,145	A	159721517	0,628
rs56292903	0,8756	0.69-1.111	0,273	0,206	0,219	A	159721534	0,591
rs72939998	0,8757	0.69-1.111	0,273	0,206	0,219	T	159721722	0,591
rs113711908	1,338	1.04-1.728	0,025	0,152	0,125	T	159721786	0,769
rs112190498	0,8539	0.668-1.09	0,205	0,241	0,255	C	159721954	0,503
rs112202751	1,338	1.04-1.728	0,025	0,152	0,125	G	159722218	0,769
rs56186252	0,8539	0.668-1.09	0,205	0,241	0,255	C	159722553	0,503
rs35901244	1,3429	1.041-1.74	0,024	0,152	0,125	T	159723905	0,752
rs55794845	0,8693	0.677-1.116	0,272	0,213	0,225	A	159724532	0,521
rs13001180	1,0078	0.741-1.372	0,960	0,132	0,132	T	159724645	0,549
rs113143827	1,3574	1.044-1.772	0,023	0,146	0,120	C	159724896	0,733
rs113266979	0,8691	0.676-1.117	0,273	0,212	0,224	A	159724996	0,519
rs35495547	1,0347	0.762-1.406	0,827	0,150	0,149	T	159725345	0,506
rs10172521	1,2412	0.982-1.571	0,071	0,296	0,274	G	159725582	0,618
rs184753966	0,8655	0.67-1.118	0,269	0,208	0,220	G	159725745	0,509
rs6711833	1,0358	0.768-1.398	0,818	0,155	0,153	A	159726323	0,514
rs192699746	0,5692	0.263-1.22	0,148	0,016	0,022	A	159727324	0,572
rs144758148	1,0817	0.435-2.776	0,866	0,013	0,013	G	159728710	0,52
rs112605456	1,3592	1.044-1.777	0,024	0,145	0,119	A	159728893	0,729
rs190418388	1,3592	1.044-1.777	0,024	0,145	0,119	C	159729290	0,729
rs145180144	0,8701	0.677-1.118	0,277	0,212	0,224	A	159732107	0,52
rs2687343	0,8683	0.673-1.121	0,278	0,211	0,223	C	159732603	0,507
rs62185467	1,1178	0.734-1.709	0,605	0,073	0,070	T	159732791	0,516
rs62185469	1,4572	1.052-2.026	0,024	0,130	0,110	C	159735154	0,525
rs62185474	1,4558	1.049-2.029	0,026	0,134	0,114	C	159739588	0,503
rs59501992	1,0793	0.427-2.815	0,872	0,012	0,011	G	159768335	0,58

rs149124199	1,0289	0.406-2.679	0,952	0,011	0,011	T	159773444	0,619
rs146880268	1,026	0.4-2.698	0,957	0,009	0,008	A	159800590	0,781
rs148503012	1,0304	0.4-2.729	0,951	0,008	0,007	A	159832857	0,871
rs147272691	0,9739	0.379-2.553	0,956	0,011	0,011	G	159837405	0,605
rs140518101	1,031	0.4-2.732	0,950	0,008	0,007	A	159909053	0,893
rs111478250	1,0591	0.42-2.749	0,903	0,013	0,013	G	159962652	0,533
rs144441965	1,0382	0.414-2.669	0,936	0,014	0,013	AG	159965018	0,513
rs78583250	1,059	0.42-2.75	0,903	0,013	0,013	G	159967904	0,533
rs142225567	1,0325	0.399-2.746	0,947	0,008	0,007	G	159979010	0,879
rs146015463	1,0362	0.4-2.764	0,942	0,007	0,007	C	159989668	0,924
rs111517052	1,0363	0.399-2.765	0,941	0,008	0,007	C	159991318	0,885
rs189752604	1,036	0.399-2.765	0,942	0,008	0,007	T	159997354	0,885
rs138427912	1,0361	0.399-2.765	0,942	0,008	0,007	G	160000451	0,885
rs113201833	1,0361	0.399-2.765	0,942	0,008	0,007	A	160001949	0,885
rs141991149	1,0364	0.399-2.766	0,941	0,007	0,007	G	160037270	0,923
rs149420847	1,0375	0.399-2.773	0,940	0,009	0,009	G	160051712	0,745
rs113514838	1,0412	0.399-2.799	0,934	0,008	0,007	A	160062155	0,873
rs144759577	1,0409	0.397-2.805	0,935	0,008	0,008	A	160077389	0,785
rs113521854	1,0427	0.397-2.82	0,932	0,010	0,009	C	160198427	0,677
rs113293804	1,0528	0.401-2.848	0,917	0,011	0,011	T	160201774	0,581
rs111980269	1,0185	0.386-2.757	0,970	0,010	0,010	T	160234675	0,618
rs74725723	1,0243	0.388-2.777	0,961	0,007	0,007	A	160247193	0,865
rs75843722	1,0243	0.388-2.777	0,961	0,007	0,007	G	160247393	0,865
rs77695483	1,039	0.399-2.781	0,937	0,012	0,012	A	160276297	0,535
rs114196637	1,0583	0.407-2.837	0,908	0,011	0,011	A	160288251	0,567
rs150793252	1,0609	0.401-2.894	0,905	0,008	0,008	A	160313236	0,782
rs76497799	1,0468	0.402-2.804	0,925	0,012	0,012	G	160319575	0,52
rs190438217	2,00E-04	0-17.985	0,828	0,000	0,001	T	160380819	0,553
rs115180277	1,0439	0.394-2.849	0,931	0,010	0,010	T	160568550	0,624
rs144244876	1,0236	0.386-2.782	0,962	0,012	0,012	G	160675886	0,522
rs145280818	1,024	0.386-2.786	0,962	0,012	0,012	T	160695375	0,521

*P value from logistic regression adjusted for age and sex including the genotype probabilities/dosages as derived from imputation to account for imputation uncertainty

Supplementary Table S4. Analyzed genomic regions and corresponding oligonucleotide primers for PCR amplification

Fragment	Position on chromosome 2 [hg19]	Fragment size [bp]	Forward primer (5' -> 3')	Reverse Primer (5' -> 3')
Promoter	159,651,088-159,651,537	450	CAATAATGGCTAAGCTTAAAGTTGT	GTGCACCTGCAATGTAACAG
Exon 1	159,651,457-159,652,045	589	AGATGTCCTGAAGCAAAGAGC	GGTAGACAGAGAAGCTTCCGT
Intron 1.1	159,652,693-159,653,272	580	CTTCCAACTGCCTTCCTTCTT	TCATTCGAACTTTCATTTGGTT
Intron 1.2	159,653,108-159,653,867	760	AGGCTCCTTACATAGACAAAGACA	TACCGGGCCTGCATTTTA
Intron 1.3	159,655,462-159,656,171	710	TGCAGGCTTATGTTTATCTTCACT	GAAGCGGAAGATAATATCCCTAGA
Intron 1.4	159,659,652-159,660,267	616	ATGTGTAGAATAACTGTGGATGA	ATACCCACCTTCCATCCAGA
Exon 2	159,660,178-159,660,965	788	GGCAGGATTATTAATGCAGTTT	GCCTTGGTTTGTCCTAAAATAACT
Intron 2.1	159,660,875-159,661,384	510	AAACAAGGTAGGGACTCTTAATTTT	CTCGGTACCTATGTGTTTCTGG
Intron 2.2*	159,661,876-159,662,944	1069 (deletion absent)<br>191 (deletion present)	CATGACAAAAGGGAAGCTGTGC	GCTATCCTCATTTATAGCCCCAAAGA
Exon 3	159,663,504-159,664,070	567	ATGTGGCCTTTTCAGTGGGA	TTCCTCCAACCCTAGACCAT
Exon 3b	159,664,059-159,664,411	353	GGGTTGGAGGAAGGCTGGGTACC	TCGAGGTGGCCGGATGGCCTG
Intron 3.1	159,667,434-159,668,027	594	CTGGGATTTGTGCTCGTTCA	TTATGACTAGAGAGCCCTGAAAT
Intron 3.2	159,667,947-159,668,395	449	ATACTACCCAACTTCAGGCTCTAA	CAGCTTTCCAGAGTATAAGCTACA
Intron 3.3	159,670,892-159,671,351	460	GTTGGCAGAGATAATCCTTGG	GGCCAGCAAGGAAGGAAT
Intron 3.4	159,671,834-159,672,072	239	TGAGGAGTTTTTCGTCCCCACTCT	TTTCTAGCCCTAAACCCATGGGAGT
Exon 4	159,672,011-159,672,594	584	AAGGACAGTAAATGAGAGAAGAC	AGGTATTCTGGATGCTTCACTT
Exon 4b	159,682,865-159,682,995	537	CCCTTTCCTTCCTTAGACCTTCC	TTGGAAGAAGATTCCTCAACTACAC
Exon rare_5	159,710,049-159,710,318	272	CCAGCATTAAGGGCTGGTTTT	GGTTGGAGAGGAAGAAGTACACG
Exon 5b	159,718,792-159,719,425	634	CGCAGACATGATGCTGGGGGT	ACATGCAAGACGGGGAATTGA

* Fragment was not resequenced but visually inspected by gel electrophoresis

Supplementary Table S5. Resequencing results of selected genomic regions (see Supplementary Table S5 and Supplementary Figure S3). Individuals were selected to be homozygous for the non-risk alleles rs17810398:C and rs17810816:A (*N*=8) or homozygous for risk alleles rs17810398:T and rs17810816:G (*N*=12). SNPs rs17810398 and rs17810816 are highlighted in green. Newly identified risk variants are highlighted in blue. The alleles are given for the forward strand.

dbSNP ID / GeneBank ID (Position)	Position on chromosome 2 [hg19]	Allele 1	Allele 2	Individuals homozygous for non-risk alleles	Individuals homozygous for either risk allele
				N(Allele 2) / N(chromosomes)	N(Allele 2) / N(chromosomes)
rs925781	159,651,734	G	A	10 / 16	0 / 24
rs1356173	159,651,973	G	A	4 / 16	0 / 24
rs12463934	159,652,852	C	T	4 / 16	0 / 24
rs7578195	159,653,250	T	C	1 / 16	0 / 24
rs71421092	159,653,626	A	G	0 / 16	5 / 22
rs12996550	159,653,721	C	G	3 / 16	0 / 22
rs6437202	159,656,114	G	C	8 / 14	0 / 16
rs6756488	159,660,277	T	A	4 / 16	0 / 24
rs75277023	159,660,494	G	A	0 / 16	24 / 24
rs11297084	159,660,646	-	A	6 / 16	0 / 24
rs17810398	159,660,870	C	T	0 / 16	24 / 24
rs2271663	159,661,077	C	T	6 / 16	0 / 24
rs147412692	159,661,111	A	G	0 / 16	9 / 24
rs11684616	159,661,318	A	G	0 / 14	0 / 22
rs6146986	159,661,997-159,662,874	-	878 bp deletion	0 / 16	24 / 24
rs9869	159,663,599	T	C	4 / 14	2 / 24
rs10497199	159,663,616	G	A	4 / 14	0 / 24
rs75859613	159,664,016	T	C	1 / 16	0 / 24
rs62183691	159,664,285	G	A	6 / 16	0 / 24
NM_001017920.2_c.207+3984 / HQ220184 (77)	159,667,611	G	A	1 / 16	0 / 24
rs908402	159,667,731	G	A	6 / 16	0 / 24
rs71421093	159,668,241	A	G	4 / 16	0 / 24
rs11693126	159,670,945	T	C	0 / 16	0 / 22
NM_001017920.2_c.208-1029 / HQ220186 (225)	159,671,188	C	T	0 / 16	1 / 22
rs17810816	159,671,992	A	G	0 / 16	24 / 24
rs1515922	159,672,168	G	A	1 / 16	0 / 24
rs61740878	159,672,252	G	A	1 / 14	0 / 24
rs10016	159,672,442	A	G	9 / 14	0 / 24
rs35919361	159,683,271	C	A	2-4 / 4*	1-2 / 2*
rs9750235	159,710,189	A	T	11 / 16	1 / 24
rs144087548	159,718,894	A	T	0 / 16	23 / 24
rs34995873	159,719,031	A	G	1 / 16	0 / 24

* Due to an extensively long AT/T-stretch, only three individuals could be resequenced after subcloning the obtained stutter bands.

Supplementary Table S6. Variants detected by cDNA re-sequencing of eight RPE/retina tissue samples heterozygous for rs17810398. Alleles are given for the + strand.

**dbSNP ID**	**RPE/retina tissue sample**							
	**ID_A**	**ID_B**	**ID_C**	**ID_E**	**ID_2**	**ID_11**	**ID_13**	**ID_14**
Gender	Female	Male	Male	Male	Male	Female	Male	Male
rs17810398	C/T	C/T	C/T	C/T	C/T	C/T	C/T	C/T
rs9869	C/T	T/T	T/T	T/T	T/T	C/T	C/T	C/T
rs10497199	G/G	A/G	A/G	G/G	A/G	A/G	G/G	G/G
rs34995873	A/A	A/A	A/A	A/A	A/A	A/A	A/A	A/G

Supplementary Table S7. Isoform-specific allele frequencies of SNP rs17810398 in 351 resequenced cDNA clones from two heterozygous RPE/retina tissue samples (ID_13, ID_14).

	**rs17810398 alleles**		***P*** *
	**C**	**T**	
Isoform 1	107	103	Reference
Isoform 2	36	41	0.60
Isoform 3	22	1	1.2×10^{-5}
Isoform 4	24	2	3.3×10^{-5}

* obtained from Fisher's exact test to test for deviations from the distribution of the reference transcript (i.e. isoform 1)
