# Supplementary figures and images for: A Candidate Gene Association Study Identifies DAPL1 as a Female-Specific Susceptibility Locus for Age-Related Macular Degeneration (AMD)
Source: Neuromolecular Med. 2015 Feb 14;17(2):111–20. doi: 10.1007/s12017-015-8342-1 (PMC4419162; doi:10.1007/s12017-015-8342-1)

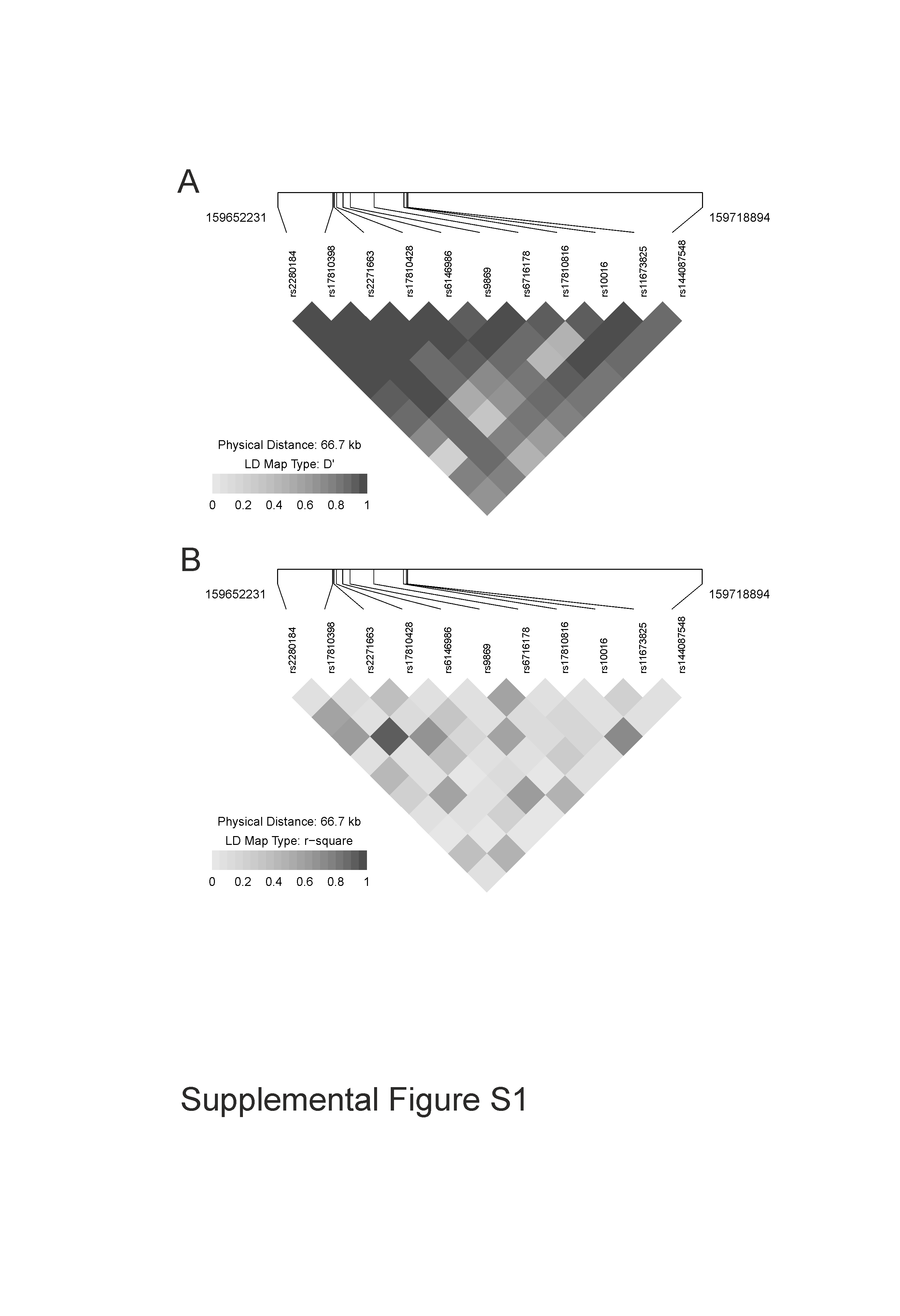

Supplement: Supplementary file 2 — Supplementary Figure S1. Linkage disequilibrium (LD) map of the DAPL1 gene locus. SNP positions are indicated by vertical/diagonal lines. A. Values of r² are indicated by coloring (white, low r²; black, high r²). B. Values of D’ are indicated by coloring (white, low D’; black, high D’). (TIFF 537 kb) [file 12017_2015_8342_MOESM2_ESM.tif]

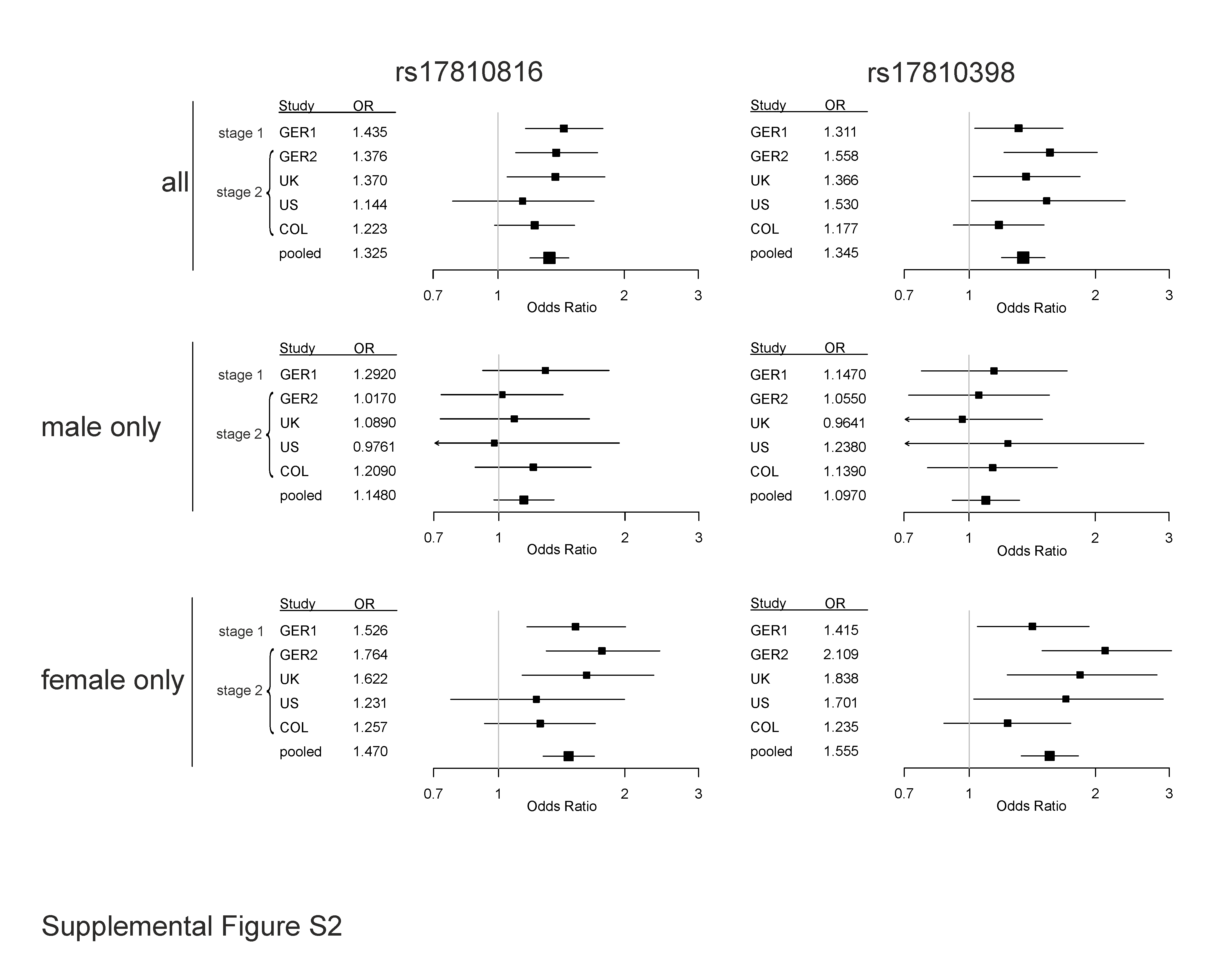

Supplement: Supplementary file 3 — Supplementary Figure S2. Sex-specific analysis in the combined study of candidate SNPs rs17810398 and rs17810816 in the DAPL1 gene. Odds ratios and corresponding 95% confidence intervals are given with the size of each rectangle representing the respective number of cases. Late stage sub-phenotypes were combined. (TIFF 818 kb) [file 12017_2015_8342_MOESM3_ESM.tif]

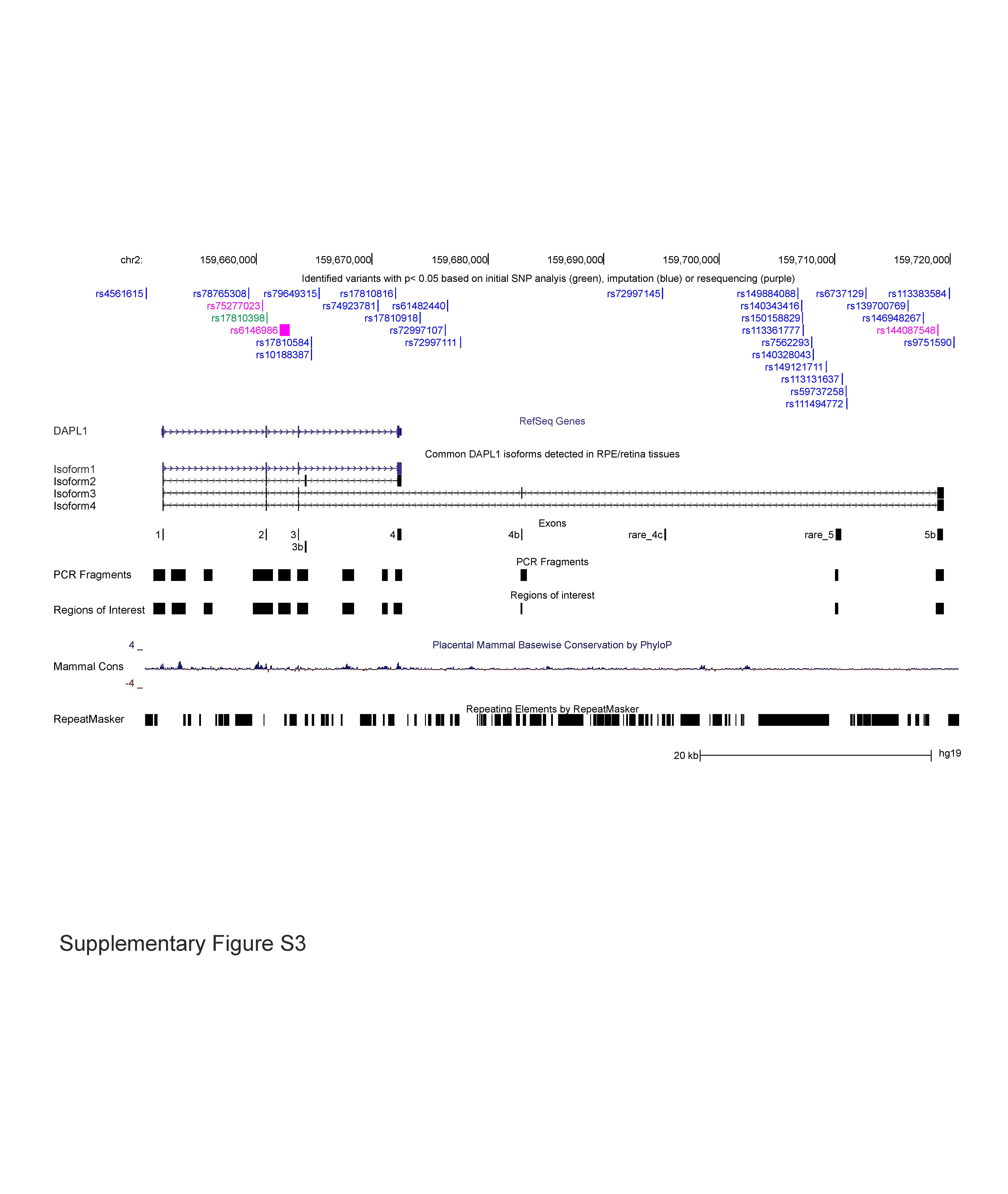

Supplement: Supplementary file 4 — Supplementary Figure S3. Resequencing strategy of the DAPL1 locus. A screenshot of the UCSC genome browser with default and custom tracks at position hg19:chr2: 159,650,435-159,721,003 is shown (http://genome.ucsc.edu). Track names are given above each track. From top to bottom, tracks are as follows: (1) Identified risk variants based upon discovery study (green), imputation analysis (blue; Supplementary Table S3) or resequencing (purple; Supplementary Table S5); (2) RefSeq sequence of DAPL1 (NM_001017920.2); (3) Common DAPL1 transcripts as identified in four RPE/retina tissue samples (isoform 1: HQ179934/NM001017920.2, isoform 2: HQ179935, isoform 3: HQ179936, isoform 4: HQ179937); (4) Exons identified in common and rare isoforms of DAPL1; (5) Resequenced PCR fragments (Supplementary Table S4); (6) Regions of interest based upon exon structure and conservation; (7) “46-Way Most Cons” track of the UCSC genome browser(14.03.2014); (8) UCSC RepeatMasker track (14.03.2014). (TIFF 1273 kb) [file 12017_2015_8342_MOESM4_ESM.tif]

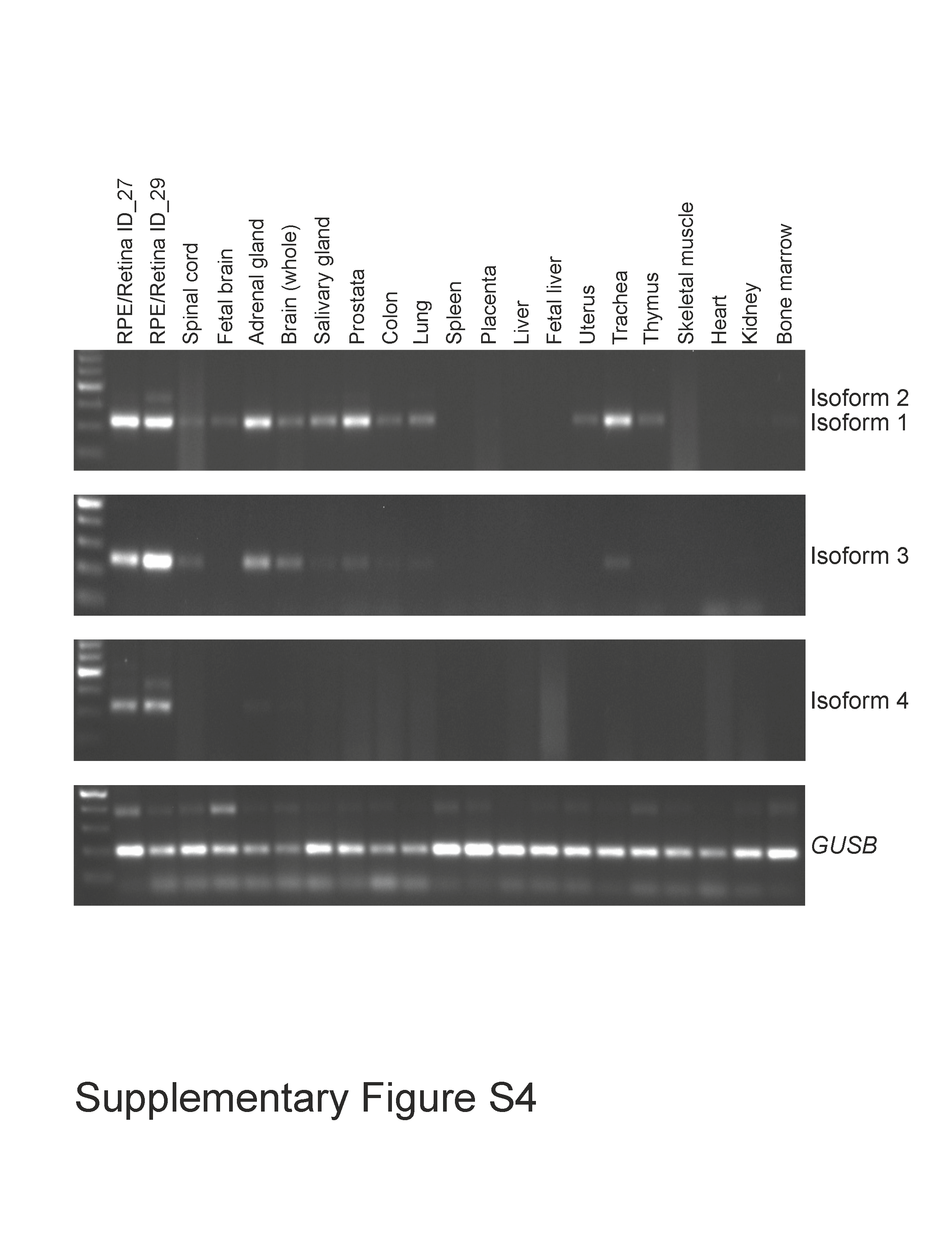

Supplement: Supplementary file 5 — Supplementary Figure S4. RT-PCR expression analysis of DAPL1 isoforms. All forward and reverse primers used were intron-spanning to avoid amplification of traces of genomic contamination in the mRNA preparations. Expected and observed fragment sizes were as follows: 329 bp (isoform 1), 448 bp (isoform2), 340 bp (isoform 3) and 331 bp (isoform 4). Expression analysis of housekeeping gene β-glucuronidase (GUSB; 197 bp) served as a control for first-strand cDNA integrity. (TIFF 1627 kb) [file 12017_2015_8342_MOESM5_ESM.tif]
